# Identification of active catalysts for the acceptorless dehydrogenation of alcohols to carbonyls

**DOI:** 10.1038/s41467-021-25214-1

**Published:** 2021-08-24

**Authors:** Tao Wang, Jin Sha, Maarten Sabbe, Philippe Sautet, Marc Pera-Titus, Carine Michel

**Affiliations:** 1 Center of Artificial Photosynthesis for Solar Fuels, School of Science, Westlake University, Hangzhou, Zhejiang Province China; 2 Eco-Efficient Products and Processes Laboratory (E2P2L), UMI 3464 CNRS – Solvay, Shanghai, China; 3 Department of Materials, Textiles and Chemical Engineering, Ghent University, Zwijnaarde, Belgium; 4 Department of Chemical and Biomolecular Engineering, University of California, Los Angeles, Los Angeles, CA USA; 5 Department of Chemistry and Biochemistry, University of California, Los Angeles, Los Angeles, CA USA; 6 Univ Lyon, ENS de Lyon, CNRS UMR 5182, Laboratoire de Chimie, Lyon, France

**Keywords:** Heterogeneous catalysis, Density functional theory, Reaction mechanisms

## Abstract

Acceptorless dehydrogenation into carbonyls and molecular hydrogen is an attractive strategy to valorize (biobased) alcohols. Using 2-octanol dehydrogenation as benchmark reaction in a continuous reactor, a library of metal-supported catalysts is tested to validate the predictive level of catalytic activity for combined DFT and micro-kinetic modeling. Based on a series of transition metals, scaling relations are determined as a function of two descriptors, i.e. the surface binding energies of atomic carbon and oxygen. Then, a volcano-shape relation based on both descriptors is derived, paving the way to further optimization of active catalysts. Evaluation of 294 diluted alloys but also a series of carbides and nitrides with the volcano map identified 12 promising candidates with potentially improved activity for alcohol dehydrogenation, which provides useful guidance for experimental catalyst design. Further screening identifies β-Mo_2_N and γ-Mo_2_N exposing mostly (001) and (100) facets as potential candidates for alcohol dehydrogenation.

## Abstract (web summary)

Identifying active catalysts for the conversion of alcohols into aldehydes or ketones and molecular hydrogen is highly desirable. Here the authors develop and validate against experiments a screening model based on DFT calculations and scaling relationships for identifying alcohol dehydrogenation catalysts.

## Introduction

The oxidation of alcohols to carbonyl compounds is one of the most fundamental and useful organic reactions for both academic research and industrial applications^1^. Traditionally, the aerobic oxidation of alcohols relying on green oxidants such as molecular oxygen and hydrogen peroxide instead of inorganic oxidants has been regarded as economically and environmentally promising^2^. Heterogeneous catalysts based on transition metals such as Pd^3^, Pt^4^, Au^5–7^, and Ru^8^ are often regarded as active and efficient for oxidation reactions. An alternative approach is to eliminate the hydrogen acceptor and produce not only a highly valuable carbonyl compound, but also H_2_^9^. Although acceptorless dehydrogenation reactions are less thermodynamically favored than oxidation reactions^10^, they limit the risk of over-oxidation to the carboxylic acid^11^. Besides, these reactions are significant for H_2_ production from biomass-based alcohols^12^, as well as for H-transfer reactions^13,14^.

In homogenous catalysis, successful catalysts for acceptorless alcohol dehydrogenation into carbonyl compounds rely on Ru^11,15^ Ir^16–18^ and Os^19^ complexes. Comprehensive reviews on the important progress of homogenously based acceptorless alcohol dehydrogenation reactions are also available^20,21^. However, such systems suffer from the common drawbacks associated to homogenous catalysis, namely complex recycling, as well as the use of additives, which hinder large-scale industrial applications. To avoid these shortcomings, heterogeneous catalysis has received high priority and a series of transition metals were tested for this reaction, for which Ru^22^, Pt^23^, Pd^24^, Re^25^, Ag^26,27^, Au^28^, Fe^29^, Mn^29^, Cu^30^, Co^29,31,32^ and Ni^33,34^, and alloys were identified as potential active catalysts, as recently reviewed by Shimizu et al.^35^.

To assist the experimental development and smart catalyst design, theoretical calculations can play a complementary and even decisive role^36,37^. In the last decade, DFT-based mechanistic investigations for alcohol dehydrogenation reactions have become easily available. For example, methanol and ethanol adsorption and dehydrogenation into CO and H_2_ have been widely studied on the surface of different transition metals such as Pt^38,39^, Pd^40^, Ir^41^, Rh^42^, Ni^43^ and Cu^44^ using both periodic slab models and metal clusters. These calculations provide useful energetic information on the alcohol decomposition pathways on each individual metal. Furthermore, Brønsted-Evans-Polanyi (BEP) correlations based on DFT studies focusing on C–H and O–H bond cleavage in alcohols have been reported for predicting activity trends for methanol^45^, ethanol^46^, and polyalcohol dehydrogenation^47^. In particular, Lausche et al.^48^. reported the dehydrogenation of CH_3_OH on the (211) surface of Cu, Ag, Pt, Pd and Rh catalysts, and formulated a descriptor-based kinetic modeling approach for predicting the catalytic activity of a library of heterogeneous catalysts.

Owing to the industrial relevance of aliphatic alcohol dehydrogenation into aldehydes and ketones, it is of great significance to find and even design efficient catalysts for this reaction. Despite the extensive experimental and theoretical investigations reported to date on transition metals, including a first-principle predictive micro-kinetic study^49^, no combined experiment-theory report is available for fast, cost-effective, and time-efficient in silico screening of metals for alcohol dehydrogenation. Herein, we present a DFT-based micro-kinetic study to predict the activity of transition metals for alcohol dehydrogenation into ketones. Our strategy focuses on building an experimentally verified activity-descriptor relation to efficiently screen different types of catalysts and provide guidance for experimental design and discovery of promising catalysts for acceptorless alcohol dehydrogenation into ketones and H_2_.

## Results and discussion

DFT calculations for the model reaction: DFT calculations were conducted to explore the alcohol dehydrogenation mechanism on a group of transition metal catalysts. Methanol (CH_3_OH) dehydrogenation into formaldehyde (CH_2_O) and H_2_ was first chosen as model reaction to avoid the intrinsic difficulties ascribed to configuration space exploration. Two pathways are a priori possible for alcohol dehydrogenation: (1) the alkoxy path CH_3_OH → CH_3_O +H → CH_2_O + 2H; and (2) the hydroxyalkyl path CH_3_OH → CH_2_OH +H → CH_2_O + 2H.

**Fig. 1:**
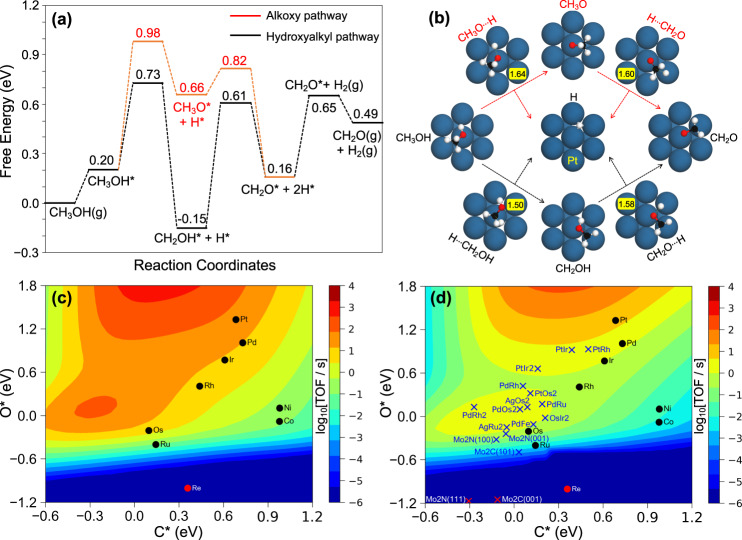
DFT calculations exploring methanol dehydrogenation into formaldehyde. Free energy profile **a** and structures of related intermediates and transition states **b** for CH_3_OH dehydrogenation on Pt(111) via the hydroxyalkyl path (in black) and the alkoxy path (in red) using molecular CH_3_OH and the pristine surface as energy reference at 453 K; **c** Turnover frequencies (TOFs) of CH_2_O production from CH_3_OH as a function of the C and O adsorption energies (referred to CH_3_OH, H_2_O and H_2_) at 453 K with *p*_*CH3OH*_ = 101 kPa (0.1% conversion), and **d** turnover frequencies (TOFs) of 2-octanone production from 2-octanol at *p*_OL_ = 101 kPa (15% conversion). (C in black, O in red, H in white, Pt in blue, numbers in the yellow box provide the length (Å) of bond being broken in TS).

As an illustrative example, Fig. 1a,b represent the free energy profiles at 453 K and the initial (IS), final (FS), and transition state (TS) structures on Pt(111) surface. CH_3_OH adsorbs on the top site with its O atom interacting with one surface Pt atom. For the alkoxy path, the O-H bond breaking in CH_3_OH to form the alkoxy CH_3_O intermediate has an energy barrier of 0.78 eV and an O–H bond length of 1.64 Å, and this step is endothermic by 0.46 eV. Further C-H bond cleavage breaking in CH_3_O to form CH_2_O has an energy barrier of only 0.16 eV and a C–H bond length of 1.60 Å, and this is exothermic by 0.50 eV. For the hydroxyalkyl path, the C–H bond breaking in CH_3_OH yields the CH_2_OH intermediate with a C–H bond length of 1.50 Å in the transition state. This step has an energy barrier of 0.53 eV and is exothermic by 0.35 eV. Further O-H bond breaking in CH_2_OH to form CH_2_O has an energy barrier of 0.76 eV with an O-H bond length of 1.58 Å in TS, and is endothermic by 0.31 eV. According to the free energy profiles, the most favorable path on Pt(111) is the hydroxyalkyl one with more stable intermediates and lower energy barriers. Adsorbate-adsorbate interactions, which can be affected by the H coverage (see potential energy diagrams in Supplementary Fig. 1), were assumed to be comparable for the different catalysts. The changes on adsorption and activation energies were calculated to be limited to 0.1 eV for the dominant hydroxyalkyl pathway (based on the degree of rate control analysis in Supplementary Tables 1 and 2), except for CH_2_O* (0.35 eV) on Pt(111) surface. As a consequence, coverage effects can impact the TOF by orders of magnitudes, but we expect the catalyst ranking to be conserved.

**Fig. 2:**
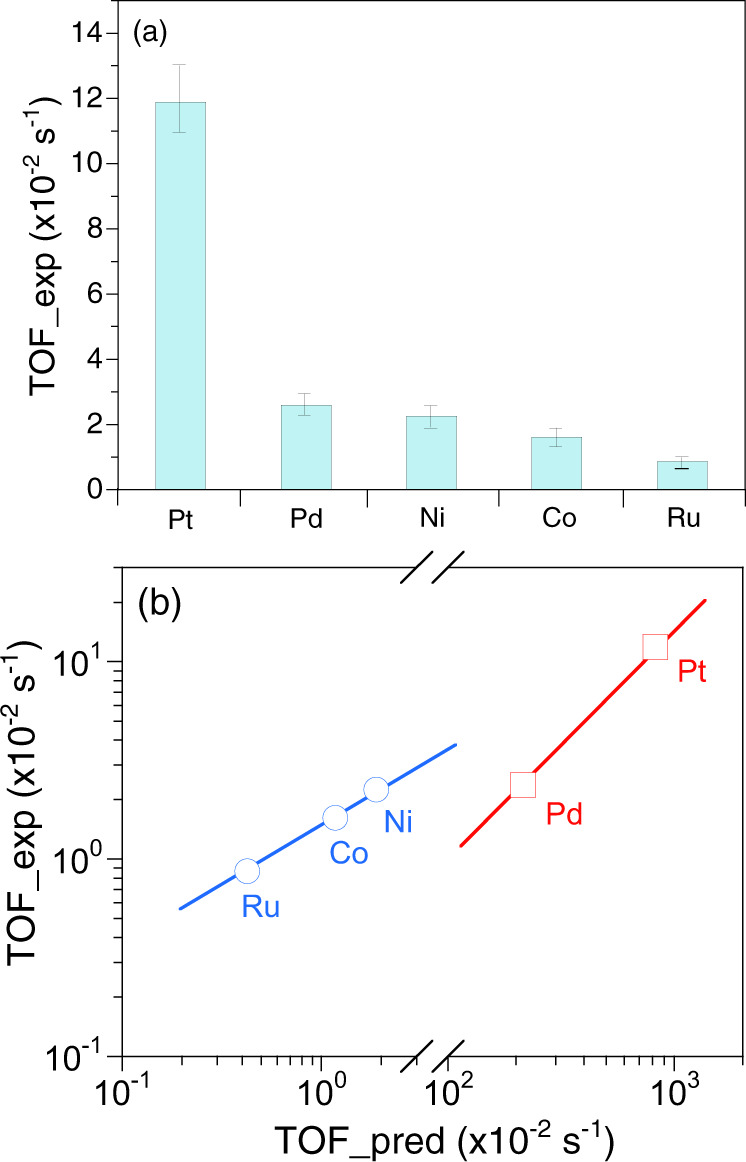
2-octanol dehydrogenation over alumina-supported metal catalysts. **a** Experimental TOF and **b** predicted TOF vs. experimental TOF. Reaction conditions: T = 453 K, P = 101 kPa, p_OL_ = 14–20 kPa, p_H2_ = 44 kPa, WHSV_OL_ = 3.3–32 h^−1^. The catalysts were pre-reduced for 4 h at different temperatures according to the H_2_-TPR profiles (Supplementary Fig. 16): 453 K for Pd/Al_2_O_3_, 473 K for Pt/Al_2_O_3_ and Ru/Al_2_O_3_, 723 K for Co/Al_2_O_3_, 773 K for Ni/Al_2_O_3_. The error bars in (**a**) refer to confidence intervals including error propagation.

**Fig. 3:**
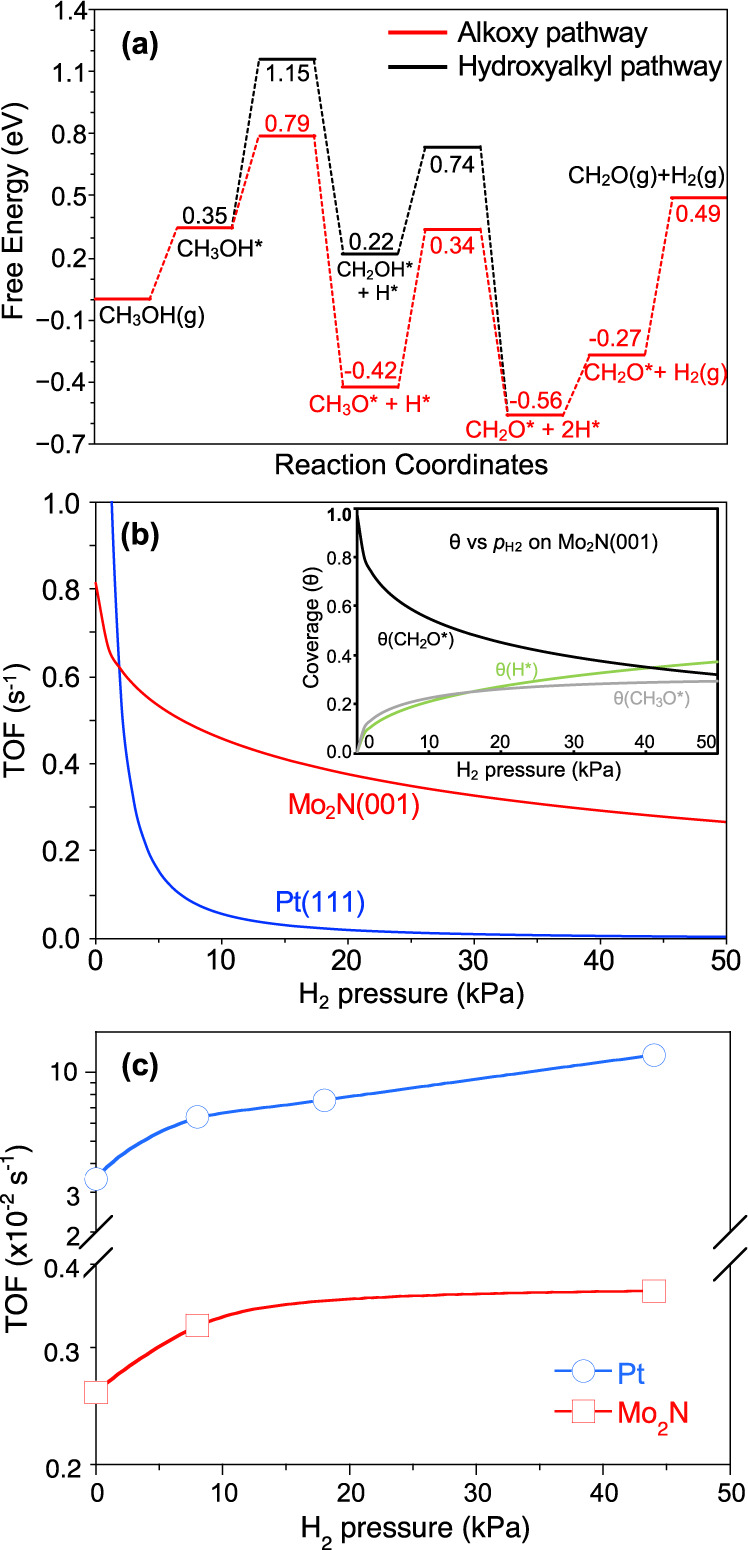
Alcohol dehydrogenation over Mo_2_N. **a** Free energy diagram for CH_3_OH dehydrogenation at 453 K on β-Mo_2_N(001) surface; **b** Predicted TOF of CH_2_O from CH_3_OH dehydrogenation on Pt(111) and β-Mo_2_N(001) surfaces at variable cofeeding H_2_ partial pressure (*p*_H2_) at 453 K with *p*_CH3OH_ = 14 kPa as well as the coverage changes of important intermediates on β-Mo_2_N (001) surface with *p*_H2_; **c** experimental evolution of the TOF for 2-octanol dehydrogenation as a function of the H_2_ partial pressure for Pt and Mo_2_N.

Analogous calculations were performed on Co(111), Rh(111), Ir(111), Ni(111), Pd(111), Os(0001), Ru(0001), and Re(0001) surfaces and the corresponding energetic and structural information are given in the supplementary Tables 3–5 and Figs. 2–11. Clearly, these metals can provide different favorable reaction paths. For example, on Pd and Ir, the hydroxyalkyl path should be the most favorable with a stable hydroxyalkyl intermediate. On the more oxophilic Ni, Co, and Ru surfaces, the alkoxy intermediate is strongly stabilized and becomes even more stable than CH_2_O, so that the alkoxy path should be the most favorable one. However, the alkoxy intermediate might also be too stable and prevent its complete dehydrogenation. In this view, a detailed micro-kinetic modeling will provide more insights into the impact of the relative stability of the intermediates and transition states on the favorable reaction pathways as shown in the following section.

General activity trends of transition metals in CH_3_OH dehydrogenation: To enable catalyst optimization and build an easily accessible descriptor for activity prediction, the CatMAP software was used to simulate maps of turnover frequency (TOF) as a function of catalyst descriptors^50^. Here, we used the adsorption energy of atomic C and O as natural and quick-to-evaluate descriptors of the overall reaction kinetics. In this method, scaling^51^ and BEP^52^ relations were applied to acquire the energetic information of the whole reaction network, which provides a basis for further micro-kinetic modeling. Based on systematic DFT simulations of CH_3_OH dehydrogenation mechanism on nine transition metals, we tested the quality of linear relationships. A MAE < 0.2 eV for all the scaling and BEP relations for this reaction provides good basis for further micro-kinetic modeling (Supplementary Table 6). To place the reaction system far from equilibrium (0.19% for CH_3_OH), a 0.1% conversion at 453 K and 101 kPa CH_3_OH was chosen as input reaction condition for kinetic modeling with CatMAP, which corresponds to *p*_CH3OH_ = 99.9 kPa and *p*_CH2O_ = *p*_H2_ = 0.1 kPa. Turnover frequencies were used to describe the activity of each catalyst. As shown in Fig. 1c, two high activity zones are observed in the volcano plot, which represent the hydroxyalkyl (above, low ΔE_O_) and alkoxy (below, strong ΔE_O_) reaction pathways, respectively. A clear activity order for the different metals can be discerned.

Validation of theoretically predicted activity trend by experimental 2-octanol dehydrogenation: Evaluating directly the agreement between micro-kinetic modeling and experimental results will provide validation of the theoretically identified activity trends of metals for CH_3_OH dehydrogenation shown in Fig. 1c, which is essential for rational catalyst screening. For better comparison with the experimental results, we modeled 2-octanol dehydrogenation on the metal catalysts by correcting the CH_3_OH/CH_2_O-to-2-octanol/2-octanone reaction free energies. For example, the overall gas-phase equilibrium constant from CH_3_OH to CH_2_O and H_2_ amounts 8.40 × 10^−5^ bar at 453 K and 101 kPa using default NASA thermochemical polynomials^53^. At this temperature, the equilibrium constant for 2-octanol derived from the same thermochemical database is much higher (7.26 × 10^−1^ bar) due to the difference between the enthalpies of formation of CH_2_O/2-octanone and CH_3_OH/2-octanol. As a result, the CH_3_OH dehydrogenation equilibrium conversion amounts only 0.19% at 101 kPa, while it becomes 56.3% for 2-octanol dehydrogenation at 101 kPa. In this view, the micro-kinetic simulations for 2-octanol dehydrogenation were computed from the DFT data for CH_3_OH dehydrogenation by multiplying the alcohol adsorption and ketone desorption equilibrium constants by the factor (7.26 × 10^−1^/8.40 × 10^−5^)^0.5^. With this correction, we built a map for 2-octanol dehydrogenation to 2-octanone at 453 K and 101 kPa as a function of C and O adsorption energies (Fig. 1d). Clearly, the trend for the different metals is similar to that of CH_3_OH (Fig. 1c) and 2-octanol (Fig. 1d) dehydrogenation, which is a good basis for further catalyst screening.

With this established activity volcano map for 2-octanol dehydrogenation, our first effort was to experimentally validate the predicted activity trend for different transition metals in Fig. 1d. To this aim, a series of transition metal catalysts was synthesized based on alumina-supported Ni, Co, Ru, Pd, and Pt catalysts, and silica-supported Pd and Pt (see SI for details). The main properties of the catalysts are listed in Supplementary Table 7, whereas the metal reducibility, dispersion and particle size distributions are compiled in Supplementary Figs. 12 and 13. The catalytic activity was measured in a continuous fixed-bed reactor at 453 K using a catalyst weight in the range of 33–430 mg and a 2-octanol flowrate in the range 1.2–2.4 mL/min. The reaction conditions were adjusted to achieve low conversion (<50%), far from chemical equilibrium. At the tested conditions, Pt exhibited the highest dehydrogenation activity per surface metal atom towards 2-octanone (1.19 × 10^−1^ s^−1^ for alumina-supported and 1.09 × 10^−1^ s^−1^ for silica supported), followed by Pd (2.38 × 10^−2^ s^−1^ for alumina-supported and 2.42 × 10^−3^ s^−1^ for silica-supported), Ni (2.26 × 10^−2^ s^−1^), Co (1.62 × 10^−2^ s^−1^) and Ru (0.87 × 10^−2^ s^−1^) (Fig. 2a, Supplementary Table 8). This trend agrees well with the prediction in Fig. 1d as illustrated in the parity plot of experimental TOF vs. predicted TOF for 2-octanol dehydrogenation (Fig. 2b). In all cases, 1-octene was observed as byproduct with a selectivity <5% for Co, Pd and Pt, and <15% for Ni and Ru (Supplementary Fig. 14). The quantitative agreement is remarkable for Ru, Co and Ni. One can note that the measured TOF for Pt and Pd are significantly lower than the calculated ones (by a factor of 100 and 30 respectively). This may be due to coking, that would decrease the number of accessible sites and accordingly the catalytic activity (Supplementary Fig. 15, Supplementary Table 8). The effect of coking is also apparent on the other metals with a coke loading always larger than 7 mol C / mol surface metal. Since the catalysts are active despite coke loading being well above a few monolayers, the amount of coke cannot be quantitatively related to a loss of active sites. Co-adsorbed H is also limiting the predicted TOF: on the very active Pt, we found this effect to yield to a drop of the predicted TOF down to 3.1×10^−2^ s^−1^ which matches the experimental TOF (see also Fig. 3b below). Overall, the jointed theoretical and experimental effort provides a good basis for extensive catalyst screening.

Predicted new catalysts: On the basis of the experimentally validated TOF map in Fig. 1d, we are able to perform an efficient screening of potential active catalysts for alcohol dehydrogenation. Following a similar strategy as in our recent work on alcohol amination^54^, we computed the C and O adsorption energies for 294 dilute alloys and evaluated their credentials for dehydrogenation (Supplementary Table 9). As shown in Supplementary Fig. 17, dilute alloys of B in A (noted AB_n_) were modeled by substituting one (n = 1) or two neighboring (n = 2) A atoms on a *p*(3×3) supercell by metal B, resulting in a B coverage of 0.11 or 0.22 ML respectively. This corresponds to single atom alloys as described in the literature^55^, or dimer-atom alloys. This procedure eventually identifies 12 out of those 294 alloys with potentially high activity in alcohol dehydrogenation as shown in the blue crosses in Fig. 1d, spanning the region of the secondary maximum in reaction rate located around E_O_=E_C_=0, since the global maximum on the map (top-left volcano peaks in Fig. 1d) lies out of the energy span covered by the considered transition metals and alloys. To our knowledge, there is no clear synthesis protocol to control those specific alloys (e.g., AgRu_2_). We believe that further state-of-the-art experimental synthesis and tests of these candidates would provide essential contributions for catalyst development targeting alcohol acceptorless dehydrogenation. Besides, the predicted candidates are mainly based on noble metals (Pt, Pd, Rh, Ru, etc.), which potentially hinders large-scale applications. If non-precious metals are targeted, a new type of active site is needed to potentially reach that region. Therefore, as first trial we focused on a series of metal carbide and nitride catalysts owing to their noble-metal-like activity in hydrotreating reactions^56^, for which close-packed model surfaces were selected. We verified that carbide and nitride catalysts follow the same scaling relations as the metal catalysts in Supplementary Fig. 18. As shown in Fig. 1d and Supplementary Table 8, the close packed surfaces of carbide and nitride catalysts such as hexagonal Mo_2_C(001), cubic γ-Mo_2_N(111) and tetragonal β-Mo_2_N(112) surfaces fall into the dark blue region on the bottom of the map due to their strong O adsorption, reflecting their potentially low activity for alcohol dehydrogenation. However, close packed surfaces are not often the most representative facets of carbide and nitride particles, and hence do not reflect entirely their catalytic properties. As a result, based on surface stability and Wulff construction information, the other exposed hexagonal Mo_2_C(101)^57^, γ-Mo_2_N(100) and β-Mo_2_N(001) surfaces were also chosen for computing the C and O adsorption energies. Overall, both C and O adsorption energies shift close to the active region, where β-Mo_2_N(001) and γ-Mo_2_N(100) with very similar surface structures (see the optimized coordinates in the Supplementary Data 1) are close to the secondary maximum of the volcano (Fig. 1d).

To further validate this prediction, the detailed CH_3_OH dehydrogenation mechanism on β-Mo_2_N(001) surface was computed, and the free energy diagram is shown in Fig. 3a. Then, a micro-kinetic model was established to compare the performance of β-Mo_2_N(001) surface with the Pt(111) surface in Fig. 3b. In the kinetic modeling, the role of H_2_ co-feeding at 453 K and *p*_CH3OH_ = 14 kPa was first considered to mimic the experimental conditions. Figure 3b reveals that the TOF of both Pt and Mo_2_N catalysts decreases at higher *p*_H2_ in the feed, and Pt(111) shows much sharper decline. As soon as H_2_ is co-fed with the alcohol, β-Mo_2_N(001) is expected to exhibit a higher TOF than Pt(111): the β-Mo_2_N(001) surface is potentially active for alcohol dehydrogenation.

With such predictions in hand, we prepared in situ a Mo_2_N sample using a reported method (see Supplementary Methods for details). The sample was highly pure and crystalline based on the β-Mo_2_N phase, with traces of metal Mo, as inferred from XRD (Supplementary Fig. 19) and HRTEM (Supplementary Fig. 20). The average particle size was 43 nm, whereas the Mo dispersion was *ca*. 2.5% by assuming a spherical particle shape. The catalytic activity was measured in a continuous fixed-bed reactor using 200 mg Mo_2_N and a 2-octanol flowrate of 1.2 mL/min and *p*_H2_ = 5.5 kPa. The 2-octanol conversion was 7% with 100% selectivity of 2-octanone. At these conditions, the dehydrogenation activity per total surface Mo was 2.85 ×10^−3^ s^−1^. This value is ~30 times lower than the calculated TOF in Fig. 1d. This can be explained by the limited ratio of (001) facets in our synthesized Mo_2_N as shown in Supplementary Fig. 20, and by contamination due to strongly bound reaction products and intermediates.

We measured the TOF for Pt and Mo_2_N at variable *p*_H2_ in the feed, and the experimental trends are provided in Fig. 3c. A low *p*_H2_ is clearly detrimental to the activity for Pt, while the activity of Mo_2_N is preserved. This apparent contradiction with the computed results suggests a drop of the number of available sites on Pt when *p*_H2_ is too low. Indeed, a high enough *p*_H2_ is necessary to maintain active sites free of coke, while the calculations assumed a constant number of Pt active sites. This is apparently less detrimental on Mo_2_N, a noticeable advantage of this catalyst. We believe that further state-of-the-art methodology will be able to synthesize phase-pure tetragonal β-Mo_2_N and cubic γ-Mo_2_N exposing mostly (001) and (100) facets, respectively, which could have higher activity in alcohol dehydrogenation.

In conclusion, to identify active catalysts for the conversion of alcohols into aldehydes or ketones and molecular hydrogen, a combined DFT and micro-kinetics approach was validated against experimental data obtained on a benchmark reaction. The reaction pathways of methanol dehydrogenation to formaldehyde and H_2_ on the close-packed surface of series transition metals (Co, Rh, Ir, Ni, Pd, Pt, Os, Ru, and Re) are systematically calculated and the TOF map as a function of C and O atoms adsorption energies is proposed with descriptor-based micro-kinetics modeling. The predicted activity trend for different metals is experimentally validated, paving the way to an *ab* initio screening of active catalysts for alcohol dehydrogenation. The two active plateaux on the volcano map with higher activity are off the range of energies spanned by the close packed transition metals under investigation. Thus, more active catalysts could be found, but not combining such metals into alloys. Evaluation of 294 dilute alloys by combining different elements in periodic table only identified 12 potentially promising candidates with comparable activity compared to  the most active Pt catalyst, which will trigger further state-of-art experimental efforts to test their performance. The screening of a series of metal carbides and nitrides revealed that tetragonal β-Mo_2_N and cubic γ-Mo_2_N exposing mostly (001) and (100) facets are potential economically viable candidates to substitute noble metals. We believe the established framework in this work lays a good basis for the discovery of novel catalyst for efficient alcohol dehydrogenation.

## Methods

Materials and methods regarding the experiments can be found in supplementary methods. All computations were performed by applying the plane-wave based density functional theory (DFT) method with the Vienna *Ab* Initio Simulation Package (VASP) and periodic slab models^58^. The electron-ion interaction was described with the projector augmented wave (PAW) method^59^, while the electron exchange and correlation energy was solved within the generalized gradient approximation with the Perdew-Burke-Ernzerhof formalism (GGA-PBE)^60^. An energy cut-off of 400 eV and a second-order Methfessel-Paxton electron smearing with σ = 0.2 eV were used to ensure accurate energies with errors less than 1 meV per atom^61^. Geometry optimization was done when forces became smaller than 0.02 eV/Å and the energy difference was lower than 10^−6^ eV. A vacuum layer of 12 Å between periodically repeated slabs was set to avoid interactions among slabs. The density-dependent dDsC method was used for the dispersion correction^62^. Nine transition metals were chosen as the model catalysts and the close-packed surfaces of each metal were used to simulate the catalytic activity, i.e., the (111)-p(3 × 3)-4L surface model of Co, Rh, Ir, Ni, Pd, Pt as well as (0001)-p(3×3)-4L surface of Ru, Os and Re. Spin-polarization was included for Ni and Co systems to correctly describe magnetic properties. A (5 × 5) k-point mesh is used for sampling the Brillouin zone. The nudged elastic band (NEB) method was applied to locate the transition states and stretching frequencies were analyzed to characterize a transition state with only one imaginary frequency^63^.

## Supplementary information

Supplementary Information

Description of Additional Supplementary Files

Supplementary Data 1

## Data Availability

The coordinates of all optimized structures generated in this study are provided as Supplementary Data 1.

## Code availability

The code used to perform this work is available at https://github.com/SUNCAT-Center/catmap under the 10.5281/zenodo.5062182.
