# Supplementary material for: Identification of active catalysts for the acceptorless dehydrogenation of alcohols to carbonyls
Source: Nat Commun. 2021 Aug 24;12:5100. doi: 10.1038/s41467-021-25214-1 (PMC8385104; doi:10.1038/s41467-021-25214-1)
Supplement: Supplementary file 1 — Supplementary Information [file 41467_2021_25214_MOESM1_ESM.pdf]

Supplementary Information

Identification of Active Catalysts for the Acceptorless Dehydrogenation of Alcohols to Carbonyls

Tao Wang,[1,*] Jin Sha,[2] Maarten Sabbe,[3] Philippe Sautet[4,5,*], Marc Pera-Titus,[2,*] Carine Michel,[6,*]

1) Center of Artificial Photosynthesis for Solar Fuels, School of Science, Westlake University, 18 Shilongshan Road, Hangzhou 310024, Zhejiang Province, China.

2) Eco-Efficient Products and Processes Laboratory (E2P2L), UMI 3464 CNRS – Solvay, 3966 Jin Du Road, Xin Zhuang Ind. Zone, 201108 Shanghai, China.

3) Department of Materials, Textiles and Chemical Engineering, Ghent University, Technologiepark125, 9052, Zwijnaarde, Belgium.

4) Department of Chemical and Biomolecular Engineering, University of California, Los Angeles, Los Angeles, CA 90095, United States.

5) Department of Chemistry and Biochemistry, University of California, Los Angeles, Los Angeles, CA 90095, United States.

6) Univ Lyon, ENS de Lyon, CNRS UMR 5182, Laboratoire de Chimie, 46 allée d'Italie, F69364 Lyon, France.

Supplementary Methods

DFT Method and Models

Methanol dehydrogenation into formaldehyde and hydrogen was chosen as a model reaction for our DFT simulations. All computations were performed by applying the plane-wave based density functional theory (DFT) method with the Vienna *Ab Initio* Simulation Package (VASP) and periodic slab models.[s1,s2] The electron ion interaction was described with the projector augmented wave (PAW) method,[s3,s4] while the electron exchange and correlation energy was solved within the generalized gradient approximation with the Perdew-Burke-Ernzerhof formalism (GGA-PBE).[s5] An energy cut-off of 400 eV and a second-order Methfessel-Paxton electron smearing with σ = 0.2 eV were used to ensure accurate energies with errors less than 1 meV per atom.[s6] Geometry optimization was done when forces became smaller than 0.02 eV/Å and the energy difference was lower than 10^{-6} eV. A vacuum layer of 12 Å between periodically repeated slabs was set to avoid interactions among slabs. The density-dependent dDsC method was used for the dispersion correction.[s7] Nine transition metals were chosen as the model catalysts and the close-packed surfaces of each metal were used to simulate the catalytic activity, i.e., the (111)-*p*(3×3)-4L surface model of Co, Rh, Ir, Ni, Pd, Pt as well as (0001)-*p*(3×3)-4L surface of Ru, Os and Re. Spin-polarization was included for Ni and Co systems to correctly describe magnetic properties. A (5×5) k-point mesh is used for sampling the Brillouin zone. The nudged elastic band (NEB) method was applied to locate the transition states and stretching frequencies were analyzed to characterize a transition state with only one imaginary frequency.[s8]

Mean-field rate equations

For the surface reactions, the rate equation for the dehydrogenation steps is

$$r_{dehydrogenation} = k_{for}\theta_{IH}\theta^{*} - k_{rev}\theta_{I}\theta_{H} \quad (1)$$

where IH and I refer to the hydrogenated dehydrogenated intermediates, respectively.

The rate coefficients for the surface reaction steps were calculated from the transition state theory as follows:

$$k_{for} = \frac{k_B T}{h}\exp\left(\frac{\Delta^{\ddagger}S}{R}\right)\exp\left(-\frac{\Delta^{\ddagger}H}{RT}\right) \quad (2)$$

The activation enthalpy $\Delta^{\ddagger}H$ and entropy $\Delta^{\ddagger}S$ were calculated from statistical thermodynamics by assuming the surface to be immobile and including only the vibrational entropy from the adsorbate frequencies.

For thermodynamic consistency, the rate coefficients for the reverse surface steps, k_{rev}, were calculated from the rate coefficients of adsorption and the equilibrium constant of the dehydrogenation reaction

$$k_{rev} = \frac{k_{for}}{K_{eq}} \quad (3)$$

The rate coefficients for the adsorption steps were assumed to be non-activated and limited to the flux of molecules to the surface as given by kinetic theory of gases

$$k_{ads} = \frac{s_0 F}{p} \quad \text{with} \quad F = \frac{p}{N\sqrt{2\pi m k_B T}} \quad (4)$$

where N is the surface density of active sites, which was taken as 10^{19} m^{-2}, a typical atom density in the surface of closest packed metals, s_0 is the initial sticking probability taken as 1 to avoid inclusion of data from experimental sources, m is the

mass of the adsorbate, p denotes the pressure and k_{ads} is the rate coefficient of adsorption expressed as adsorption rate per active site in units of bar^{-1} s^{-1}.

The adsorption rate was expressed as follows

$$r_{ads,i} = k_{ads,i}\, p_i\, \theta^* \quad (5)$$

where p_i is the pressure of the adsorbate in the gas phase and θ^* is the fraction of free active sites. The rate coefficient is equal to the flux of molecules to the surface as given by kinetic theory of gases.

For molecular desorption, the rate coefficient was expressed as a function of the adsorption equilibrium, K_{ads}, and rate coefficient for adsorption, to ensure thermodynamic consistency

$$k_{des} = \frac{k_{ads}}{K_{ads}} \quad (6)$$

Dissociative adsorption ($H_{2(g)}$ + 2* → 2H*) was also considered to be non-activated. Accordingly, the rate equation can be expressed as follows

$$r_{ads,diss} = 2k_{ads,diss}\, p_{H_2}\, \theta^{*2} \quad (7)$$

Construction of the micro-kinetic model

A micro-kinetic model was developed for the set of CH_3OH dehydrogenation reactions with the following elementary steps:

**CH_3OH dehydrogenation**
$CH_3OH(g) + 2^* \leftrightarrow CH_3OH^* + {}^*$
$CH_3OH^* + {}^* \leftrightarrow CH_3O^* + H^*$
$CH_3O^* + {}^* \leftrightarrow CH_2O^* + H^*$
$CH_3OH^* + {}^* \leftrightarrow CH_2OH^* + H^*$
$CH_2OH^* + {}^* \leftrightarrow CH_2O^* + H^*$
$2H^* \leftrightarrow H_2(g) + 2^*$
$CH_2O^* \leftrightarrow CH_2O(g) + {}^*$

g: gas phase

*: active site

The dehydrogenation and hydrogenation steps were modeled as Horiuti-Polanyi elimination/addition of single hydrogen atoms. The production rate of the surface intermediate IH_i (s^{-1}) are given by

$$R_{IH} = \frac{d\theta_{IH}}{dt} = k_{for,1}\theta_{IH2}\theta^* - k_{rev,1}\theta_{IH}\theta_H - k_{for,2}\theta_{IH}\theta^* - k_{rev,2}\theta_I\theta_H \quad (8)$$

The site balance comprises all surface species, with i=1 to n summing from CH_3OH to CH_2O over all dehydrogenated intermediates

$$\theta^* + \theta_H + \Sigma_{i=1}^{n}\theta_{IH} = 1 \quad (9)$$

The pseudo-steady-state surface concentrations were obtained transiently by solving the differential equations in Eq. 9 using the LSODA integration routines from the ODEPACK Fortran library as implemented in the Python package SciPy until steady state was reached. The CatMAP software[s9] was used to calculate maps of turnover frequency (TOF) as a

function of catalyst descriptors, C and O in this work. Some modifications were conducted to the original software to obtain steady-state coverages, because the default approach in CatMAP tends to yield incorrect zero activity for some descriptor combinations space. This is due to the fact that CatMAP uses a Gibbs distribution of coverages as an initial guess for the micro-kinetic solver, which can lead in some cases to erroneous results. In particular, for very stable species, their predicted coverages by the Gibbs distribution can approach ~1, poisoning effectively the catalyst surface in such a way that no reactive species can adsorb. In such cases, the software immediately identifies a non-active steady state. To address this problem, a transient microkinetic simulation was performed for all grid points considered by CatMAP until a time of 10 s was reached. The resulting coverages were consecutively loaded into CatMAP and used as initial values in the default CatMAP steady state microkinetic solver. This approach ensured the correct TOF was obtained, particularly in cases where multiple steady states were possible.

Degree of Rate Control

To determine how strong the energies of transition states and intermediates control the reaction rate, the degree of rate control was analyzed according to the method by Campbell *et al.*[s10,s11,s12] In this method, the degree of rate control $X_{RC,i}$ for elementary step *i* is defined as:

$$X_{RC,i} = \frac{k_i}{r}\left(\frac{\partial r}{\partial k_i}\right)_{K_i} = \left(\frac{\partial \ln r}{\partial \ln k_i}\right)_{K_i} \tag{10}$$

where k_i is the rate constant, and r is the overall reaction rate. The equilibrium constant (K_i) is kept unchanged, which means that k_i (forward) and k_{-i} (reverse) are both varied with the same factor, which corresponds to varying the energy of the transition state. In this work, k_i and k_{-i} are multiplied by 1.1 for the analysis. Similarly, the degree of thermodynamic rate control $X_{TRC,\,n}$ of intermediate *n* is expressed as:

$$X_{TRC,n} = \frac{1}{r}\left(\frac{\partial r}{\partial\left(\frac{-G_n^0}{RT}\right)}\right) = \frac{k_n}{r}\left(\frac{\partial r}{\partial k_n}\right) = \left(\frac{\partial \ln r}{\partial \ln k_n}\right) \tag{11}$$

In which $G°_n$ is Gibbs energy of the intermediate; the expression is in practice evaluated by varying the rate coefficients for the reactions that consume this intermediate *n*, in practice usually the $k_{i,rev}$ and $k_{i+1,for}$. In the equations, all rate coefficients that are varied at the same time by varying the Gibbs energy of the intermediate are denoted k_n. To evaluate the degree of rate control, these rate coefficients are multiplied by 1.1 as well. In both cases, the stronger X differs from 0 for given step indicates a bigger influence of its rate constant on the overall reaction rate: if it is positive, it means that decreasing the Gibbs energy of the state increases the rate of the reaction, corresponding to increasing or decreasing the rate coefficients for resp. rate control of reactions and thermodynamic rate control of the intermediate. For a negative X, the opposite holds. The DRC analysis of different metals are given in Supplementary Table1-2 and Supplementary Figure 11c.

Effect of adsorbate-adsorbate interaction.

The influence of adsorbate-adsorbate interactions is a concern in any DFT/microkinetic study. It was considered on two representative catalysts, Pt and Mo_2N. For Pt, our kinetic model indicates adsorbed hydrogen as the most abundant surface

intermediate, we therefore added 6 hydrogen atoms to the used p(3x3) supercell (shown in **Supplementary Figure 1**) leading to co-coverage of 67% of hydrogen and performed all DFT calculations for CH_3OH dehydrogenation at high H coverage. Then, microkinetic modeling was re-done to compare the TOF at low and high H coverage. In these calculations we only evaluated the effect on the electronic energy, and the entropy is assumed unchanged at low and H coverage. The potential energy diagrams for methanol dehydrogenation Pt(111) surface at both low and high H coverage is shown in **Supplementary Figure 1b**, changes on the adsorption energies and activation energies are limited to 0.1 eV for the dominant hydroxyalkyl pathway (based on the degree of rate control analysis in Supplementary Table**1-2**), except for CH_2O^* (0.35 eV). For the methoxy path the differences are up to 0.25 eV on the barriers, but this path contributes little to the total TOF on Pt(111). The barriers increase in all cases, as can be expected for a dehydrogenation reaction at increased hydrogen coverage, which will bring the TOF down. We then performed microkinetic modeling based on two groups of DFT data at low and high H coverage. Despite the small changes on the electronic energies, at 0.1% conversion the TOF with the 'high H coverage' data is about 360 times smaller than using the original 'low H coverage' data (0.031 s^{-1} compared to 11 s^{-1}). The effect of coverage on the TOF is large at first sight, but similar effects can be expected for all catalysts with a similar high activity, since the hydrogen adsorption energy is rather constant over all catalysts. A reduction of the TOF will also be present for less active catalysts, but the difference can be expected to be less drastic. Therefore, the relative activity of the catalysts can be expected to remain unchanged upon inclusion of the adsorbate-adsorbate interactions.

For the Mo_2N surface, the most abundant surface species is CH_2O^*, and therefore all calculations are done with one extra CH_2O^* species co-adsorbed with the active species or transition state. Based on the degree of rate control analysis shown in Supplementary Table**1** the methoxy path is the dominant pathway and CH_3OH is the key intermediate based on the degree of thermodynamic rate control analysis in Supplementary Table**2**. For the Mo_2N catalyst, we therefore only calculated the effect of CH_2O on the methoxy pathway. Our results show that CH_3OH adsorption is almost unchanged by adding one CH_2O on the surface. (-0.39 eV at low coverage vs -0.42 eV at high coverage). The energy barrier for the rate-determining step ($CH_3OH \rightarrow CH_3O$) only increased by 0.03 eV from 0.61 eV at low coverage to 0.64 eV at high coverage. The effect of these small changes on the kinetics is negligible.

In summary, there clearly is an effect of coverage on the kinetics, as expected. The changes on the reaction barriers are limited, however, the overall picture on the turnover frequency amounts to several orders of magnitude on Pt. Due to the similar bond strength of hydrogen on the different materials, this effect is expected to be similar for all high-activity materials, and not affecting the order of activity of the different materials considered.

Experimental details

Materials

γ-Al_2O_3 (Puralox Sasol Scca-5/170, 154 m^2/g) and SiO_2 (Evonik, Sipernat2200 , 190 m^2/g) were used as supports for catalyst synthesis as received. Nickel(II) nitrate hexahydrate $Ni(NO_3)_2 \cdot 6H_2O$ (98% purity), cobalt(II) nitrate hexahydrate $Co(NO_3)_2 \cdot 6H_2O$ (98% purity), 2-octanol (99% purity) and 2-octanone (99% purity) were purchased from Sinopharm Group Co, Ltd. Ruthenium chloride $RuCl_3$ (45-55% Ru), palladium(II) nitrate dihydrate $Pd(NO_3)_2 \cdot 2H_2O$, (~40% Pd), chloroplatinic acid hexahydrate $H_2PtCl_6 \cdot 6H_2O$ (≥37.50% Pt) and MoO_3 was supplied by Sigma-Aldrich. Silicon carbide (100 mesh) was acquired from STREM. N_2 was supplied by Air Liquide (purity 99.99%). H_2 was obtained from a H_2 generator from (ANPEL, LGH-500 T). Biphenyl (99.5% purity), supplied by J&K Scientific, was used as internal standard for GC calibration. All the reactants were used as received without further purification.

Catalyst preparation

The metal-supported catalysts were prepared by incipient wetness impregnation (IWI) over γ-Al_2O_3 and SiO_2 using an aqueous solution of the metal precursor targeting 5 wt% nominal loading. In a typical preparation, the given amount of metal precursor (*e.g.*, 500 mg for 5 wt% Co) was dissolved in 1.96 g of distilled water and sonicated for 10 min at room temperature. The solution was added dropwise to 2.0 g of either γ-Al_2O_3 or SiO_2 under mixing. The mixture was kept at room temperature for 2 h, dried at 393 K for 12 h and calcined at 673 K for 4 h using a heating rate of 2 $K \cdot min^{-1}$ under static air. The catalysts were further reduced under H_2 flow [30 mL(STP)$\cdot min^{-1}$] at the desired temperature according to the H_2-TPR profiles.

The Mo_2N sample was prepared according to the protocol described in ref.[S13] The commercial MoO_3 sample (as received) was subjected to a temperature-programmed treatment under 15% v/v N_2/H_2 flow [58 mL(STP)$\cdot min^{-1}$] from room temperature to 933 K, and this temperature was kept during 18 h, resulting in an enriched β-Mo_2N catalyst.

Catalyst characterization

The bulk metal composition of the catalysts was analyzed by inductively coupled plasma (ICP) on an Activa (Horiba Jobin-Yvon) optical emission spectrometer. The dried and ground sample (~10 mg) was dissolved in 1.5 mL of concentrated *aqua regia* and 250 µL of a 48% hydrofluoric acid (HF) solution. The solution was heated at 493 K and sonicated for 30 min.

The particle size distribution was measured by HR-TEM on a JEOL, JEM-2100 (200 kV) microscope equipped with a LaB_6 electron gun. The images were analyzed by ImageJ software. At least 200 particles were counted for the statistic chart. In the analyses, we assumed that the metal particles are spherical in shape and we took explicitly into account the density ratio between the oxide and metal phases. The average particle size (surface weighted, $d_p[3,2]$) was estimated from the particle size distribution using the expression

$$d_p[3,2] = \frac{\sum_{i=1}^{i=n} d_{p,i}^3 n_i}{\sum_{i=1}^{i=n} d_{p,i}^2 n_i} \quad (10)$$

The metal dispersion, D, was measured from the average particle size according to the universal expression provided in ref[S14] and by a Supplementary Tablesuming cubo-octahedron shape.[S15]

The reducibility of the metal species in the catalysts was characterized by temperature-programmed reduction (H_2-TPR) using a Micromeritics AutoChem II 2920 instrument. The system was equipped with a thermal conductivity detector (TCD) to monitor the changes in the gas composition and a cold trap before the detector to remove vapors. H_2-TPR profiles were measured from 303 K to 1173 K using a heating rate of 10 $K \cdot min^{-1}$ under a 10% v/v H_2-Ar flow [40 mL(STP)/min]. The H_2 profiles were recorded using Autochem II software and the H_2-consumption was measured after band integration.

The phases present in the catalysts were analyzed by powder X-ray diffraction (PXRD). The PXRD patterns were recorded on a Rigaku D/Max-2200/PC diffractometer provided with Cu-Kα radiation (λ = 1.5418 Å) and a beam voltage of 45 kV. The spectra were collected in the 2θ range 10-90° with a step change of 0.02°. The patterns were indexed using the Joint Committee on Powder Diffraction (JCPDS) database and interpreted using MDI JADE 5.0 software.

The amount of coke generated on the catalysts during the dehydrogenation reaction was characterized by thermogravimetric analysis using a TGA-DSC analyzer from Mettler Toledo. The spent catalysts, recovered after 30 min on stream, were treated from 323 K to 1073 K at a heating rate of 10 $K \cdot min^{-1}$ under a 20% v/v O_2-N_2 mixture with 30

cm^3(STP)/min flowrate. The weight loss ascribed to coke oxidation was identified by the exothermic DSC peak and the weight loss attributed to water was corrected using the thermal profile measured on an Al_2O_3 blank sample subjected to the same temperature treatment.

Catalytic tests

The catalytic dehydrogenation tests were carried out in a continuous fixed-bed reactor (stainless steel, i.d. 4 mm) equipped with a liquid and gas distribution system. The inlet gas flowrates were adjusted with thermal mass flow controllers (±1% accuracy, Seven Star Electronics), while the liquid flowrate was adjusted with a liquid metering pump (±3% accuracy, Beijing Satellite Manufacturing). The temperature was regulated using PID controllers (±1 K accuracy, MTI Corporation). The fixed-bed reactor was loaded from the bottom to the top in three layers. The first layer (bottom, 10 mL) of 200-μm SiC ensured the positioning of the catalyst within the isothermal zone. The catalyst (500 mg) was loaded as a second layer followed by a third layer (upper, 10 mL) of 200-μm SiC promoting alcohol vaporization and feed mixing. The different layers were separated using glass wool.

Before the reaction, the catalysts were reduced *in situ* in the fixed bed at the desired temperature (453 K for Pd/Al_2O_3, 473 K for Pt/Al_2O_3 and Ru/Al_2O_3, 723 K for Co/Al_2O_3, 773 K for Ni/Al_2O_3) under a 50%v/v H_2-N_2 flow mixture [60 mL(STP)/min] using a 5 K·min^{-1} heating ramp and held at this temperature for 4 h. After activation, the temperature was decreased to the desired value under a 1:1 H_2-N_2 flow mixture at 101 kPa total pressure. Then, the reactant mixture was injected and the system was stabilized for 0.5-1 h. The catalytic tests were conducted at 453 K, 101 kPa total pressure, 0.6-4.0 mL.h^{-1} 2-octanol flowrate (14-20 kPa) and 30-500 mg catalyst in the presence of H_2 (44 kPa) using N_2 as carrier gas. Dedicated tests for 12 h on stream for Pt catalyst (most active) revealed no apparent change of catalytic activity, pointing out a lack of long-time catalyst deactivation by coking. The catalytic tests were conducted at least three times every 20 min on consecutive experiments with a time on stream of 3 h for each test. Preliminary tests confirmed the absence of external mass transfer effects on the catalytic activity. The reactor outlet was cooled down to room temperature and liquid samples were recovered every hour. The weight-hourly space velocity of the reactor referred to 2-octanol ($WHSV_{OL}$) and the total catalyst loading was defined as follows

$$WHSV_{OL}\left(h^{-1}\right)=\frac{F^0_{OL}\left(mL.h^{-1}\right)}{W\left(g\right)}\rho_{OL}\left(g.mL^{-1}\right) \quad (12)$$

where F^0_{OL} is the 2-octanol liquid flow at the reactor inlet, W is catalyst loading and ρ_{OL} is the density of 2-octanol at 298 K (0.82 g.mL^{-1}).

The recovered liquid samples were analyzed offline using an Agilent 7890A GC equipped with a 30 m x 0.32 mm x 0.25 μm Zebron HP-5 column with 5 wt.% phenyl groups and a FID detector. The column was programmed with a 3 K·min^{-1} initial ramp from 353 K to 373 K followed by a 50 K·min^{-1} ramp to 573 K, holding this temperature for 3 min. The 2-octanol conversion, as well as the selectivity and yield towards the 2-octanone (ONE) and 1-octene (ENE) (neither 2-octene and the ether by-product were observed), were calculated by interpolation of the corresponding calibration curves using biphenyl as internal standard as follows

$$Conversion\left(\%\right)=1-\frac{n^{out}_{OL}}{n^{in}_{OL}} \quad (13)$$

$$Selectivity_{ONE}\left(\%\right)=\frac{n^{out}_{ONE}}{n^{in}_{OL}-n^{out}_{OL}}\ ;\ Selectivity_{ENE}\left(\%\right)=\frac{n^{out}_{ENE}}{n^{in}_{OL}-n^{out}_{OL}} \quad (14, 15)$$

$$\text{Yield}_{\text{ONE}}\left(\%\right)=\frac{n_{\text{ONE}}^{\text{out}}}{n_{\text{OL}}^{\text{in}}}\ ;\ \text{Yield}_{\text{ENE}}\left(\%\right)=\frac{n_{\text{ENE}}^{\text{out}}}{n_{\text{OL}}^{\text{in}}} \qquad (16, 17)$$

where n_{OL}^{in} and n_{OL}^{out} refer to the inlet and outlet 2-octanol molar flows, respectively, n_{ONE}^{out} and n_{ENE}^{out} are the outlet molar flows of 2-octanone and 1-octene, respectively.

The turnover frequencies with respect to 2-octanol conversion and yield of 2-octanone (TOF_X and TOF_Y, respectively) were computed as follows:

$$\text{TOF}_{\text{ONE}}=\frac{w_{\text{ONE}}^{\text{out}}}{n_M \bar{D}_M \text{EOR}} \qquad (18)$$

where n_M refers to the moles of metal catalyst, D_M is the dispersion as obtained from particle size analysis, and EOR is the extent of reduction determined from temperature programmed reduction (H_2-TPR).

Supplementary Tables

Supplementary Table 1. Degree of rate control X_{RC} of each elementary step.

	X_{RC}				
**primary steps**	Pt	Pd	Ni	Co	$Mo_2N(001)$
$CH_3OH^* + * \leftrightarrow CH_3O^* + H^*$	0.00	0.00	**0.70**	**0.96**	**0.86**
$CH_3O^* + * \leftrightarrow CH_2O^* + H^*$	0.00	0.00	0.05	0.02	0.014
$CH_3OH^* + * \leftrightarrow CH_2OH^* + H^*$	0.09	0.06	0.02	0.00	0.00
$CH_2OH^* + * \leftrightarrow CH_2O^* + H^*$	**0.77**	**0.68**	0.18	0.00	0.00

Supplementary Table 2. Degree of thermodynamic rate control X_{TRC} for all the intermediates.

	X_{TRC}				
**intermediates**	Pt	Pd	Ni	Co	$Mo_2N(001)$
H	−0.57	−0.64	−0.80	0.54	-0.06
CH_3OH	0.00	0.00	0.00	0.00	0.43
CH_2OH	−0.51	0.00	0.00	0.00	0.00
CH_3O	0.00	0.00	−0.11	−1.60	0.00
CH_2O	0.00	0.00	0.00	0.00	0.01

Supplementary Table 3. Adsorption energy of all the species related to CH_3OH dehydrogenation on different catalyst surfaces.

	**H**	**CH_3OH**	**CH_3O**	**CH_2OH**	**CH_2O**
Co(111)	-0.58	-0.53	-3.41	-2.26	-1.03
Rh(111)	-0.57	-0.58	-2.92	-2.56	-1.10
Ir(111)	-0.44	-0.57	-2.59	-2.57	-0.88
Ni(111)	-0.62	-0.56	-3.25	-2.31	-1.02
Pd(111)	-0.68	-0.50	-2.50	-2.51	-0.81
Pt(111)	-0.50	-0.50	-2.20	-2.71	-0.66
Ru(0001)	-0.64	-0.65	-3.35	-2.57	-1.25
Re(0001)	-0.80	-0.66	-3.68	-2.66	-1.51
Os(0001)	-0.57	-0.72	-3.10	-2.69	-1.25
$Mo_2N(001)$	-0.44	-0.46	-3.34	-2.47	-1.62
Mo2C(101)	-0.58	-0.64	-3.46	-2.74	-1.64

Supplementary Table 4. Activation energy barriers of CH_3OH dehydrogenation to CH_2O on different catalyst surfaces

Catalyst	E_{a1} / eV	E_{a2} / eV	E_{a3} / eV	E_{a4} / eV
Co(111)	0.81	0.91	0.94	0.60
Rh(111)	0.77	0.62	0.58	0.70
Ir(111)	0.69	0.70	0.63	0.68
Ni(111)	0.82	0.74	0.88	0.65
Pd(111)	0.92	0.54	0.54	0.72
Pt(111)	0.80	0.61	0.22	0.81
Ru(0001)	0.74	0.80	0.74	0.78
Re(0001)	0.65	0.93	0.97	0.50
Os(0001)	0.73	0.84	0.61	0.83
Mo_2N(001)	0.54	0.87	0.78	0.68
Mo_2C(101)	0.89	0.90	0.96	0.86

E_{a1}: $CH_3OH \rightarrow CH_3O + H$; E_{a2}: $CH_3OH \rightarrow CH_2OH + H$; E_{a3}: $CH_3O \rightarrow CH_2O + H$; E_{a4}: $CH_2OH \rightarrow CH_2O + H$

Supplementary Table 5. Rate constants for forward (k_{for}) and reverse (k_{rev}) reactions of each elementary step during CH_3OH dehydrogenation at 453 K on different metals

Elementary steps	Co		Rh		Ir	
	k_{for}	k_{rev}	k_{for}	k_{rev}	k_{for}	k_{rev}
$CH_3OH(g) + 2^* \leftrightarrow CH_3OH^* + ^*$	2.19E+08	9.26E+09	2.19E+08	6.54E+08	2.19E+08	1.82E+10
$CH_3OH^* + ^* \leftrightarrow CH_2OH^* + H^*$	1.04E+03	6.06E+03	5.06E+06	9.85E+05	3.97E+06	1.76E+06
$CH_2OH^* + ^* \leftrightarrow CH_2O^* + H^*$	7.58E+07	2.17E+02	3.73E+07	1.02E+04	1.03E+07	3.96E+07
$CH_3OH^* + ^* \leftrightarrow CH_3O^* + H^*$	1.84E+05	5.61E-03	3.88E+05	1.45E+04	4.11E+06	3.57E+08
$CH_3O^* + ^* \leftrightarrow CH_2O^* + H^*$	1.15E+04	6.30E+06	3.40E+07	4.85E+04	1.39E+06	2.70E+04
$2H^* \leftrightarrow H_2(g) + 2^*$	2.43E+02	8.75E+08	1.19E+02	8.75E+08	1.29E+05	8.75E+08
$CH_2O^* \leftrightarrow CH_2O(g) + ^*$	7.10E+05	2.26E+08	3.25E+05	2.26E+08	2.67E+08	2.26E+08

Elementary steps	Ni		Pd		Pt	
	k_{for}	k_{rev}	k_{for}	k_{rev}	k_{for}	k_{rev}
$CH_3OH(g) + 2^* \leftrightarrow CH_3OH^* + ^*$	2.19E+08	6.76E+09	2.19E+08	5.68E+09	2.19E+08	1.66E+10
$CH_3OH^* + ^* \leftrightarrow CH_2OH^* + H^*$	8.85E+05	3.86E+06	1.70E+07	2.96E+04	1.31E+07	1.55E+03
$CH_2OH^* + ^* \leftrightarrow CH_2O^* + H^*$	1.18E+08	3.27E+02	6.66E+06	1.10E+05	3.45E+04	9.46E+07
$CH_3OH^* + ^* \leftrightarrow CH_3O^* + H^*$	3.38E+05	2.52E-01	6.18E+03	9.11E+04	2.04E+04	2.25E+09
$CH_3O^* + ^* \leftrightarrow CH_2O^* + H^*$	2.05E+04	3.31E+05	4.65E+07	9.11E+01	1.67E+11	4.92E+05
$2H^* \leftrightarrow H_2(g) + 2^*$	4.46E+01	8.75E+08	7.14E-01	8.75E+08	1.10E+03	8.75E+08
$CH_2O^* \leftrightarrow CH_2O(g) + ^*$	2.04E+06	2.26E+08	2.57E+08	2.26E+08	5.48E+09	2.26E+08

Elementary steps	Ru		Os		Re	
	k_{for}	k_{rev}	k_{for}	k_{rev}	k_{for}	k_{rev}
$CH_3OH(g) + 2^* \leftrightarrow CH_3OH^* + ^*$	2.19E+08	4.89E+08	2.19E+08	2.94E+08	2.19E+08	5.22E+08
$CH_3OH^* + ^* \leftrightarrow CH_2OH^* + H^*$	2.89E+05	5.57E+03	9.89E+04	2.41E+03	7.93E+03	2.58E-01
$CH_2OH^* + ^* \leftrightarrow CH_2O^* + H^*$	2.81E+06	1.09E+01	1.20E+06	3.82E+02	9.12E+08	6.31E-01
$CH_3OH^* + ^* \leftrightarrow CH_3O^* + H^*$	8.69E+06	2.57E+00	4.37E+06	7.69E+03	7.24E+06	4.53E-05
$CH_3O^* + ^* \leftrightarrow CH_2O^* + H^*$	7.67E+05	1.93E+05	1.07E+07	4.70E+04	6.50E+03	2.33E+01
$2H^* \leftrightarrow H_2(g) + 2^*$	6.05E+00	8.75E+08	1.47E+02	8.75E+08	7.01E-03	8.75E+08
$CH_2O^* \leftrightarrow CH_2O(g) + ^*$	6.73E+03	2.26E+08	1.73E+04	2.26E+08	1.87E+00	2.26E+08

Supplementary Table 6. TSS(IS), TSS(IS), BEP relations of each C-H and O-H bond breaking on nine transition (E_{IS}: initial state energy, E_{FS}: final state energy, E_{TS}: transition state energy, Ea: activation energy barrier, Er: reaction energy, MAE: mean absolute error)

Elementary steps	TSS/IS	TSS/FS	BEP
$CH_3OH \rightarrow CH_3O + H$	$E_{TS} = 1.68E_{IS} + 1.18$ (MAE = 0.04)	$E_{TS} = 0.12E_{FS} + 0.29$ (MAE = 0.19)	$E_a = 0.03E_r + 0.78$ (MAE = 0.05)
$CH_3OH \rightarrow CH_2OH + H$	$E_{TS} = -0.1E_{IS} + 0.10$ (MAE = 0.08)	$E_{TS} = 0.16E_{FS} + 0.27$ (MAE = 0.08)	$E_a = 0.14E_r + 0.76$ (MAE = 0.11)
$CH_3O \rightarrow CH_2O + H$	$E_{TS} = 0.63E_{IS} + 0.37$ (MAE = 0.08)	$E_{TS} = 0.78E_{FS} + 0.57$ (MAE = 0.10)	$E_a = 0.75E_r + 0.74$ (MAE = 0.09)
$CH_2OH \rightarrow CH_2O + H$	$E_{TS} = 0.99E_{IS} + 0.69$ (MAE = 0.08)	$E_{TS} = 0.37E_{FS} + 0.32$ (MAE = 0.11)	$E_a = 0.19E_r + 0.73$ (MAE = 0.05)

Supplementary Table 7. Metal content, average particle size, metal dispersion and extent of reduction for the catalysts used in this study

Entry	Catalyst	Loading (wt%) (Metal)[(a)]	Average Particle Size (nm)[(b)]	Dispersion (%)[(c)]	EOR (%)[(d)]
1	Al_2O_3	-	-	-	-
2	Ni	4.8% (4.7%)	4.2 ± 1.1	35% (32%)	100%
3	Co	4.6% (4.8%)	3.8 ± 2.0	38% (26%)	100%
4	Pd	4.9% (5.5%)	5.4 ± 1.7	27% (28%)	100%
5	Pt	4.7% (5.4%)	0.88 ± 0.23	100% (91%)	100%
6	Ru	4.4% (5.1%)	3.3 ± 1.4	44% (32%)	100%
7	SiO_2	-	-	-	-
8	Pd[e]	4.7% (6.0%)	5.2 ± 2.2	22% (28%)	100%
9	Pt[e]	4.0% (4.5%)	3.4 ± 0.9	30% (41%)	100%

[a] Determined by ICP-OES, in parentheses, value measured by H_2-TPR; [b] Determined by HR-TEM; [c] Determined from HR-TEM microscopy using the universal expression provided in ref [1], in parentheses value measured by assuming a cubooctahedron shape using ref [1]; [d] Determined from H_2-TPR: 10 $K \cdot min^{-1}$ up to 1173 K using a 10% v/v H_2-Ar mixture [40 cm^3(STP)/min]. [e] Catalysts prepared by the IWI method over SiO_2

Supplementary Table 8. Experimental results on 2-octanol dehydrogenation for γ-Al_2O_3 supported metal catalysts.[a]

Entry	Cat	Conversion (2-OL)	Selectivity (2-ONE)	Selectivity (1-ENE)	Yield (2-ONE)	Mass balance	TOF(x0.01 s^{-1})[b]	Coke (mol C / mol M_s)[c]
1	Al_2O_3	28%	-	64%	-	92%	-	-
2	Ni	45%	85%	15%	39%	99%	2.26	7.36
3	Co	47%	98%	1.8%	46%	105%	1.62	9.92
4	Pd	38%	100%	-	38%	104%	2.38	14.2
5	Pt	41%	99%	1.3%	40%	106%	11.90	7.67
6	Ru	27%	88%	12%	24%	99%	0.87	18.4
7	SiO_2	0%	-	-	-	-	-	-
8	Pd[d]	2.9%	100%	-	2.9%	98%	0.24	15.1
9	Pt[d]	19%	100%	-	19%	99%	10.93	9.36

[a] Reaction conditions: 453K, P=101 kPa, p_{OL} = 14-20kPa, p_{H2} = 44kPa, $WHSV_{OL}$=3.3-32h^{-1}. The catalysts were pre-reduced during 4 h at different temperatures according to the corresponding H_2-TPR profiles (**Supplementary Figure 15**): 453 K for Pd/Al_2O_3, 473 K for Pt/ Al_2O_3 and Ru/ Al_2O_3, 723 K for Co/ Al_2O_3, 773 K for Ni/ Al_2O_3. [b] Maximum error for TOF about 5%. [c] Measured by TGA on he spent catalysts in the temperature range 673 – 724 K (see profiles in **Supplementary Figure 14**). [d] Catalysts prepared by the IWI method over SiO_2.

Supplementary Table 9. Adsorption energies (eV) of C and O atoms on 294 different alloys as well as the carbide and nitride surfaces (referred to CH_3OH, H_2O and H_2 molecules)

Alloy	EC	EO	Alloy	EC	EO	Alloy	EC	EO	Alloy	EC	EO
AgFe	1.09	-0.17	AgFe2	0.04	-1.02	OsAu	0.34	-0.07	RhPd	0.34	0.36
AgFe2	0.04	-1.02	AgOs	0.47	-0.54	OsAu2	0.48	0.13	RhPd2	0.3	0.31
AgMo	0.81	-1.73	AgOs2	0.09	0.13	OsCo	0.06	-0.3	RhPt	0.36	0.41
AgMo2	1.11	-0.37	AgRe	0.39	-1.61	OsCu	0.16	-0.21	RhPt2	0.36	0.43
AgOs	0.47	-0.54	AgRu2	-0.05	-0.15	OsCu2	0.36	0.02	RhRe	0.57	-0.08
AgOs2	0.09	0.13	AuCo2	-0.22	-0.57	OsIr	0.21	-0.17	RhRe2	0.75	-0.42
AgRe	0.39	-1.61	CoCu	-0.49	-1.6	OsIr2	0.21	-0.02	RhRu	0.44	0.21
AgRe2	0.82	-0.1	CoGa2	0.32	-0.51	OsMn	-0.07	-0.66	RhRu2	0.62	0.05
AgRu	0.92	0.17	CoRu2	0.48	-0.39	OsPd	0.22	-0.17	RuAg	0.32	-0.28
AgRu2	-0.05	-0.15	CoSn	-0.36	-1.49	OsPd2	0.37	0.06	RuAg2	0.47	-0.1
AuCo	1.27	0.81	CuCo2	0.16	-0.65	OsPt	0.27	-0.12	RuAu	0.38	-0.24
AuCo2	-0.22	-0.57	CuFe2	0.25	-0.86	OsPt2	0.31	0.12	RuAu2	0.52	-0.03
CoAg	0.87	-0.21	CuW2	0.43	-1.26	OsRh	0.17	-0.2	RuCu	0.25	-0.27
CoAg2	1.03	0	Fe	0.06	-0.76	OsRh2	0.26	0.04	RuCu2	0.27	-0.32
CoCu	-0.49	-1.6	IrCo	0.35	0.32	OsRu	0.14	-0.21	RuIr	0.27	-0.32
CoGa	0.95	-0.15	IrCo2	0.19	-0.08	OsZn	0.28	-0.1	RuIr2	0.27	-0.29
CoGa2	0.32	-0.51	IrFe	0.51	0.35	PdFe	0.13	-0.11	RuOs	0.19	-0.39
CoOs2	0.93	7.04	IrFe2	0.29	-0.16	PdFe2	-0.15	-0.62	RuPd	0.27	-0.34
CoPd	0.91	-0.24	IrOs2	0.46	0.37	PdMo	1.1	-0.48	RuPd2	0.37	-0.16
CoPd2	1	-0.13	IrRe2	0.45	-0.19	PdMo2	0.32	-1.05	RuPt	0.31	-0.3
CoPt	0.99	-0.07	IrRu2	0.49	0.3	PdOs	0.17	-0.13	RuPt2	0.37	-0.15
CoPt2	1.08	0.01	Mo	0.03	-1.52	PdOs2	0.04	0.1	RuRh	0.24	-0.36
CoRh	0.88	-0.02	OsAg	0.29	-0.09	PdRe	0.62	-0.56	RuRh2	0.26	-0.28
CoRh2	0.87	0.04	OsAu	0.34	-0.07	PdRe2	0.17	-0.44	ZnCo	1.42	-0.21
CoRu	0.58	-0.33	OsAu2	0.48	0.13	PdRh	0.06	0.42	ZnCo2	0.76	-0.38
CoRu2	0.48	-0.39	OsCo	0.06	-0.3	PdRh2	-0.27	0.13	ZnMn	1.07	-0.33
CoSn	-0.36	-1.49	OsCu	0.16	-0.21	PdRu	0.19	0.17	ZnMn2	0.31	-0.8
CoZn	1.13	-0.15	OsCu2	0.36	0.02	PdRu2	0.02	-0.02	ZnNi	1.93	-0.05
Cu	2.75	0.77	OsIr	0.21	-0.17	PtFe	0.36	0.42	ZnNi2	1.25	-0.36

CuCo	1.15	-0.02	OsIr2	0.21	-0.02	PtFe2	0.06	-0.38	ZnOs	1.08	-0.23
CuCo2	0.16	-0.65	OsMn	-0.07	-0.66	PtMo	1.05	-0.34	ZnOs2	0.56	-0.2
CuFe	1	-0.35	OsPd	0.22	-0.17	PtMo2	0.57	-0.59	ZnRe	0.99	-0.24
CuFe2	0.25	-0.86	OsPd2	0.37	0.06	PtOs	0.39	0.27	ZnRe2	0.39	-0.47
CuMo	0.9	-1.53	OsPt	0.27	-0.12	PtOs2	0.11	0.32	RhCu2	0.06	0.17
CuMo	1.07	-1.28	OsPt2	0.31	0.12	PtRe	0.7	-0.3	RhFe	0.35	-0.02
CuMo2	1.01	-0.62	OsRh	0.17	-0.2	PtRe2	0.28	-0.11	RhFe2	0.15	-0.46
CuOs	0.83	-0.06	OsRh2	0.26	0.04	PtRh	0.5	0.93	RhMn	0.26	-0.2
CuOs2	0.69	0.28	OsRu	0.14	-0.21	PtRh2	0.21	0.53	RhMn2	0.09	-0.77
CuRe	0.76	-1.16	OsZn	0.28	-0.1	PtRu	0.47	0.68	RhOs	0.43	0.17
CuRe2	0.87	-0.15	PdFe	0.13	-0.11	PtRu2	0.33	0.25	RhOs2	0.33	0.12
CuRh	1.52	0.69	PdFe2	-0.15	-0.62	PtSc	0.73	0.53	RhPd	0.34	0.36
CuRh2	0.79	0.56	PdMo2	0.32	-1.05	PtSc2	0.84	-0.87	RhPd2	0.3	0.31
CuRu	1.16	0.22	PdOs	0.17	-0.13	ReAg	0.34	-0.79	RhPt	0.36	0.41
CuRu2	0.57	0.14	PdOs2	0.04	0.1	ReAg2	0.34	-0.96	RhPt2	0.36	0.43
CuW	0.92	-1.86	PdRe2	0.17	-0.44	ReAu	0.53	-0.82	RhRu	0.44	0.21
CuW2	0.43	-1.26	PdRh	0.06	0.42	ReAu2	0.43	-0.94	RuAg	0.32	-0.28
Fe	0.06	-0.76	PdRh2	-0.27	0.13	ReCu	0.3	-1.2	RuAg2	0.47	-0.1
GaFe	1.02	-1.08	PdRu	0.19	0.17	ReCu	0.33	-0.93	RuAu	0.38	-0.24
GaFe2	1.15	-1.13	PdRu2	0.02	-0.02	ReCu2	0.26	-1.13	RuCu	0.25	-0.27
GaNi	1.74	-0.5	PtFe	0.36	0.42	ReIr	0.61	-0.93	RuCu2	0.27	-0.32
GaNi2	1.74	-0.44	PtFe2	0.06	-0.38	ReIr2	0.63	-1.01	RuIr	0.27	-0.32
IrCo	0.35	0.32	PtOs	0.39	0.27	RePd	0.5	-0.85	RuIr2	0.27	-0.29
IrCo2	0.19	-0.08	PtOs2	0.11	0.32	RePd2	0.41	-0.47	RuOs	0.19	-0.39
IrFe	0.51	0.35	PtRe2	0.28	-0.11	RePt	0.63	-0.88	RuPd	0.27	-0.34
IrFe2	0.29	-0.16	PtRh	0.5	0.93	RePt2	0.52	-0.98	RuPd2	0.37	-0.16
IrMo	0.77	0.16	PtRh2	0.21	0.53	ReRh	0.56	-0.9	RuPt	0.31	-0.3
IrMo2	0.58	-0.73	PtRu	0.47	0.68	ReRh2	0.52	-1.04	RuPt2	0.37	-0.15
IrOs	0.59	0.58	PtRu2	0.33	0.25	ReZn	0.24	-1.15	RuRh	0.24	-0.36
IrOs2	0.46	0.37	ReAg	0.34	-0.79	RhCu	0.22	0.28	RuRh2	0.26	-0.28
NiAu2	1.33	0.46	RhMn2	0.09	-0.77	NiPt2	1.57	0.41	NiAg2	1.12	0.3
NiCo	1.02	0.05	RhMo	0.72	-0.19	NiRe	0.92	-0.48	NiAu	1.24	0.31
NiCu	1.06	0.13	RhMo2	0.64	-0.91	NiRe2	0.69	-0.54	ZnMn2	0.31	-0.8

NiCu2	1.03	0.15	RhOs	0.43	0.17	NiRh	1	0.28	ZnRe2	0.39	-0.47
NiFe	1.28	0.02	RhOs2	0.33	0.12	NiRh2	0.95	0.32	RhCu2	0.06	0.17
NiFe2	1.23	-0.18	ReCu	0.33	-0.93	NiRu	0.85	0.04	RhFe	0.35	-0.02
NiGa	1.02	0.07	ReCu	0.3	-1.2	NiRu2	0.67	-0.07	ReAg2	0.34	-0.96
NiGa2	1.04	1.47	ReCu2	0.26	-1.13	NiZn	1	0.09	ReAu2	0.43	-0.94
NiIr	0.97	0.38	RePd	0.5	-0.85	NiZn2	0.96	1.53	IrRe	0.66	0.33
NiIr2	0.97	0.39	RePd2	0.41	-0.47	OsAg	0.29	-0.09	IrRe2	0.45	-0.19
NiMo	1.19	-0.59	ReZn	0.24	-1.15	OsAg2	0.53	0.08	PtIr	0.39	0.92
NiMo2	0.84	-1.11	RhCu	0.22	0.28	RhFe2	0.15	-0.46	PtIr2	0.16	0.66
NiOs	0.75	-0.1	IrRu	0.6	0.56	RhMn	0.26	-0.2	NiAg	1.09	0.21
NiOs2	0.67	0.01	IrRu2	0.49	0.3	NiPt	1.34	0.33	MoCu2	-0.82	-2.93
NiPd	1.16	0.22	NiPd2	1.22	0.32	β-Mo_2N(001)	-0.06	-0.24	WN(111)	-0.08	-1.89
Mo_2C(001)	-0.51	-2.15	Mo_2C(101)	0.03	-0.51	γ-Mo_2N(100)	-0.12	-0.32	γ-Mo_2N(101)	0.67	-1.24
TiN(111)	-0.5	-3.31	VN(111)	0.49	-2	β-Mo_2N(112)	-0.31	-2.08	NbN(111)	0.21	-2.48

Supplementary Figures

Supplementary Figure 1. Structures and PES for CH_3OH dehydrogenation at low and high coverage on Pt(111) surface.

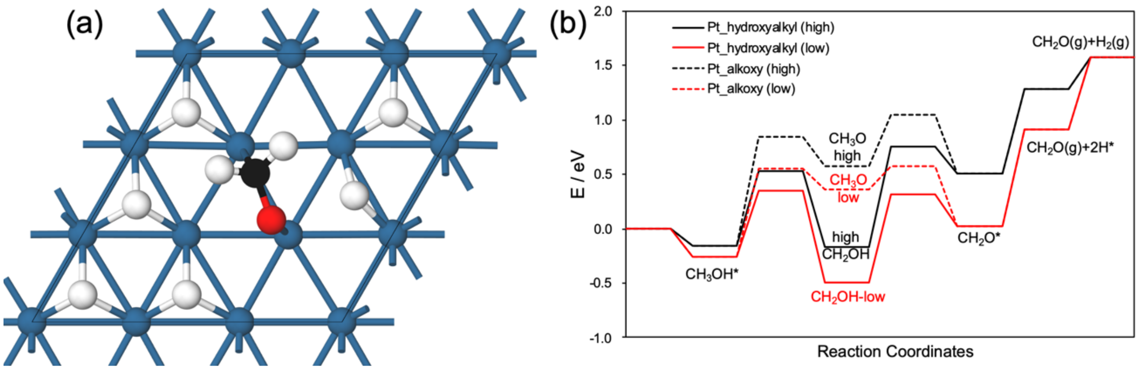

Supplementary Figure 2. Configurations of CH_3OH dehydrogenation related intermediates and transition states on Ni(111) surface (the numbers in the yellow box is the length (Å) of breaking bond in TS)

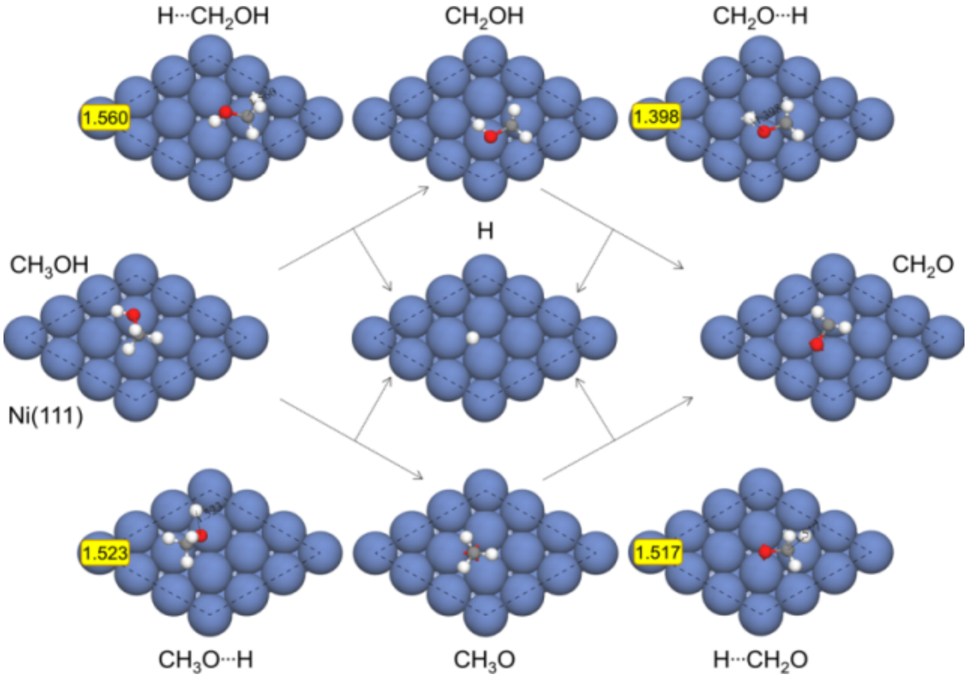

Supplementary Figure 3. Configurations of CH_3OH dehydrogenation related intermediates and transition states on Pd(111) surface (the numbers in the yellow box is the length (Å) of breaking bond in TS)

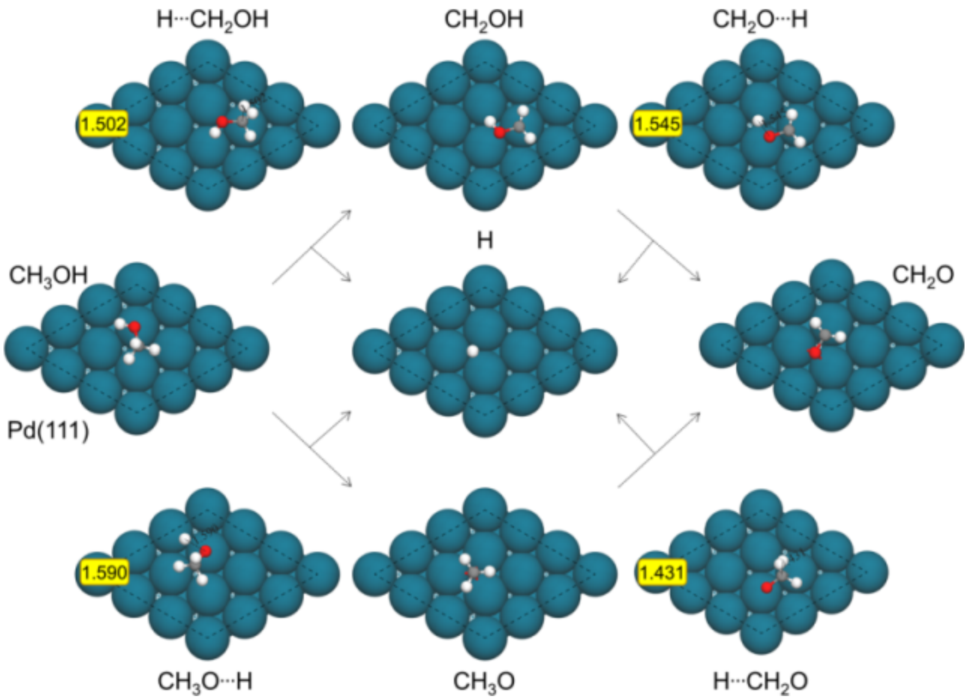

Supplementary Figure 4. Configurations of CH_3OH dehydrogenation related intermediates and transition states on Co(111) surface (the numbers in the yellow box is the length (Å) of breaking bond in TS)

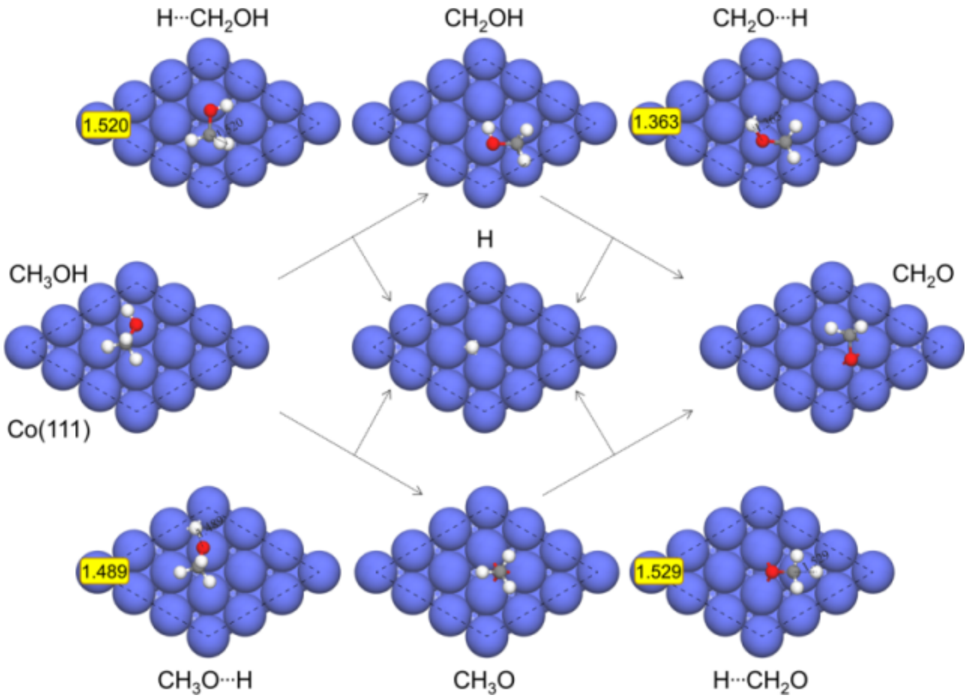

Supplementary Figure 5. Configurations of CH_3OH dehydrogenation related intermediates and transition states on Rh(111) surface (the numbers in the yellow box is the length (Å) of breaking bond in TS)

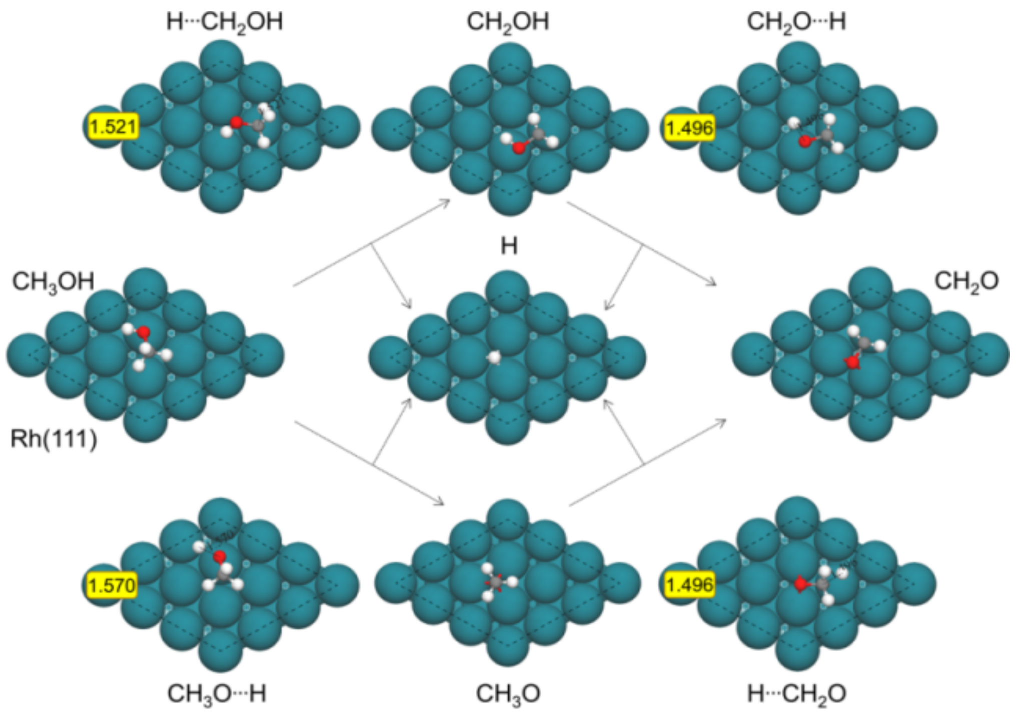

Supplementary Figure 6. Configurations of CH_3OH dehydrogenation related intermediates and transition states on Ir(111) surface (the numbers in the yellow box is the length (Å) of breaking bond in TS)

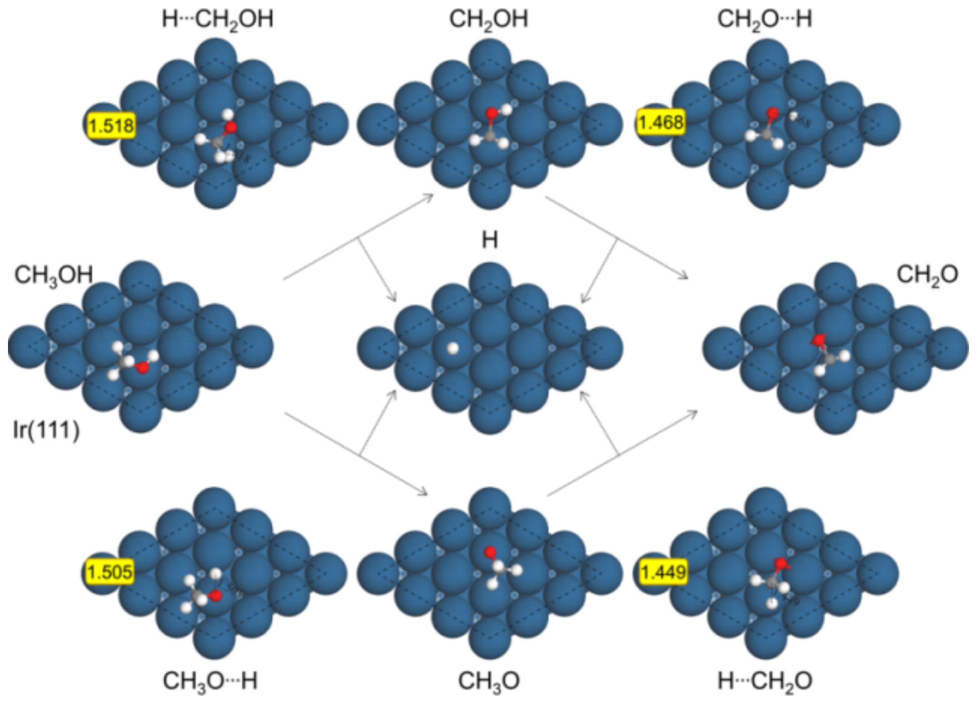

Supplementary Figure 7. Configurations of CH_3OH dehydrogenation related intermediates and transition states on Ru(0001) surface (the numbers in the yellow box is the length (Å) of breaking bond in TS)

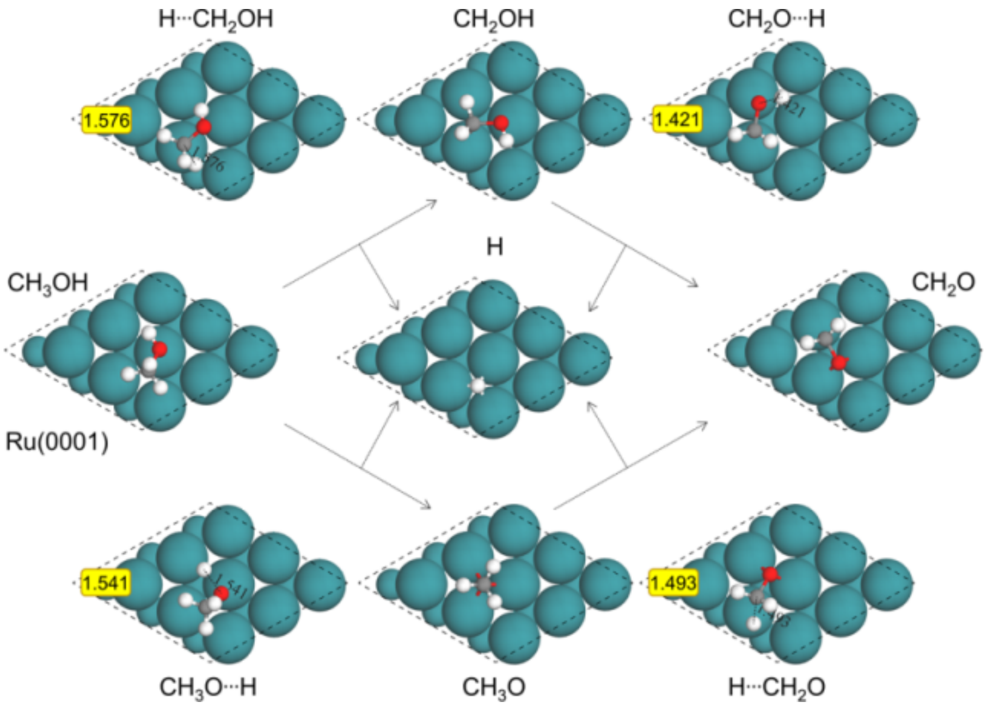

Supplementary Figure 8. Configurations of CH_3OH dehydrogenation related intermediates and transition states on Os(0001) surface (the numbers in the yellow box is the length (Å) of breaking bond in TS)

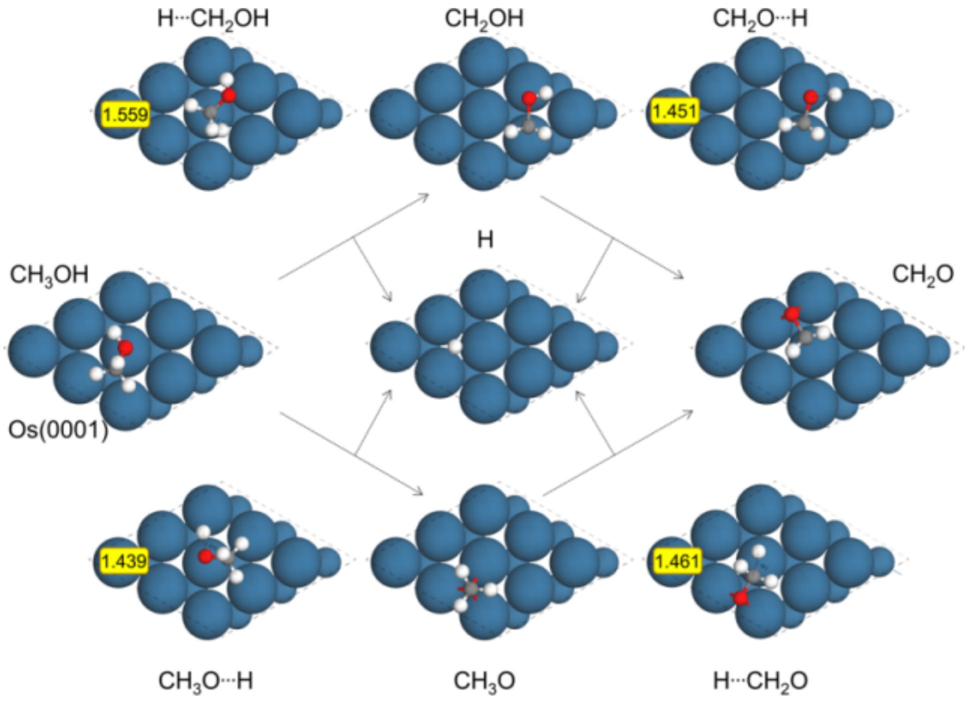

Supplementary Figure 9. Configurations of CH_3OH dehydrogenation related intermediates and transition states on Re(111) surface (the numbers in the yellow box is the length (Å) of breaking bond in TS)

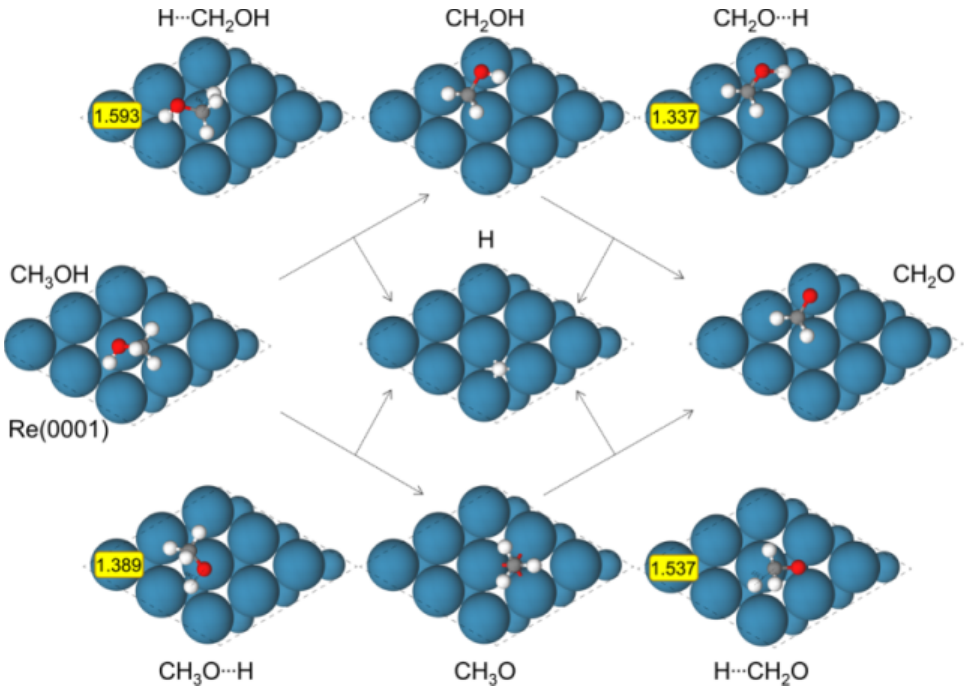

Supplementary Figure 10. Gibbs free energy diagrams of CH_3OH dehydrogenation into $CH_2O + H_2$ on the different metals at 453 K.

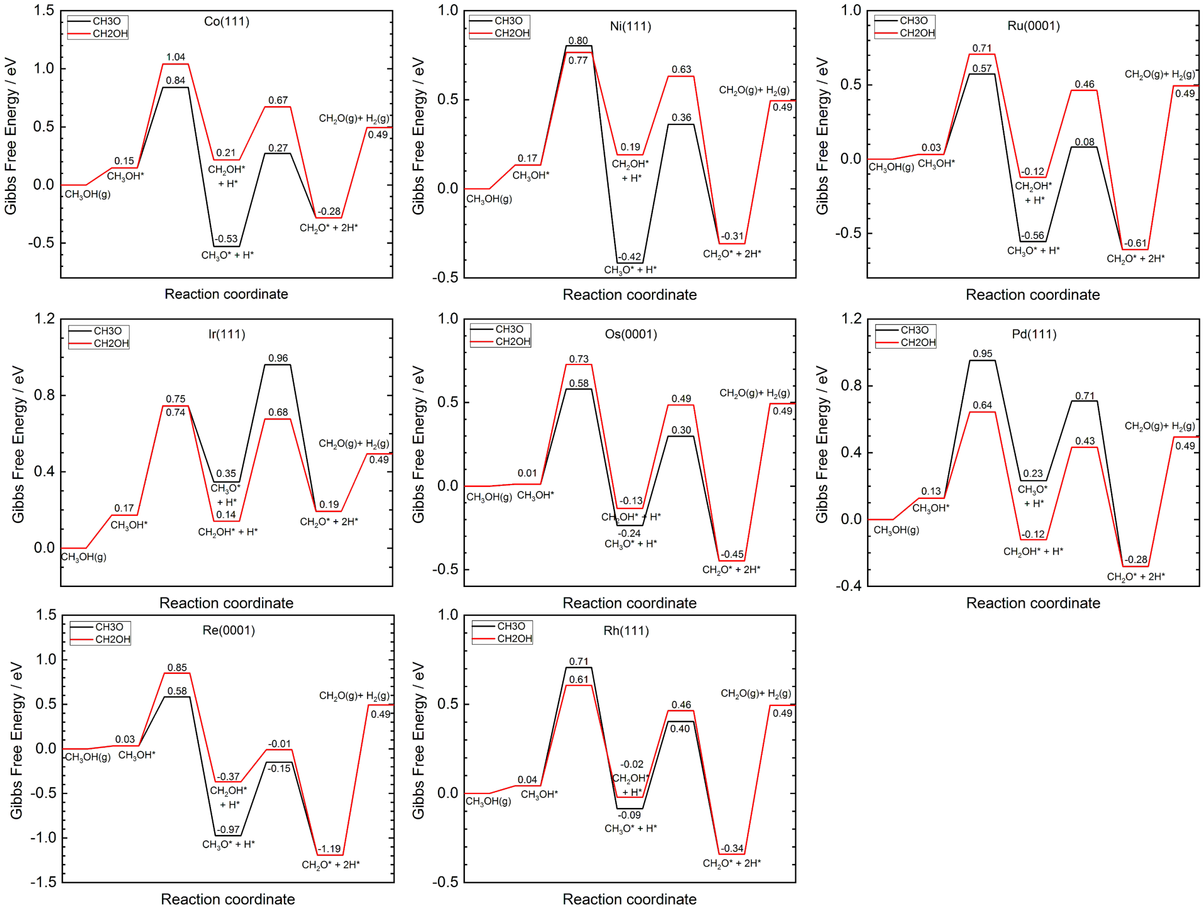

Supplementary Figure 11. TOF maps of the CH_3OH → CH_2OH +H → CH_2O + 2H pathway and CH_3OH → CH_3O +H → CH_2O + 2H pathway as well as other information from micro-kinetics modeling.

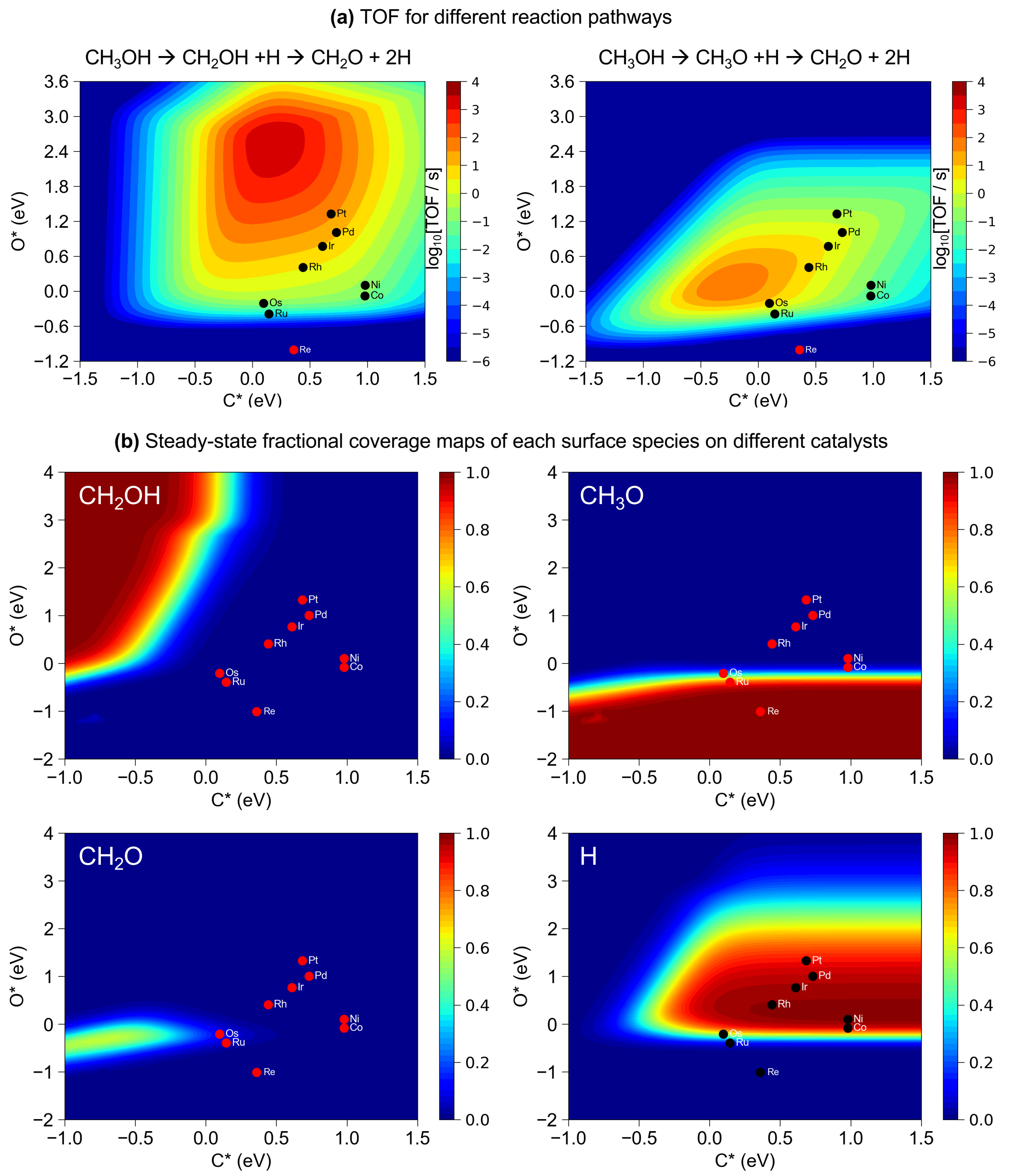

(c) Degree of rate control map for CH_3OH dehydrogenation.

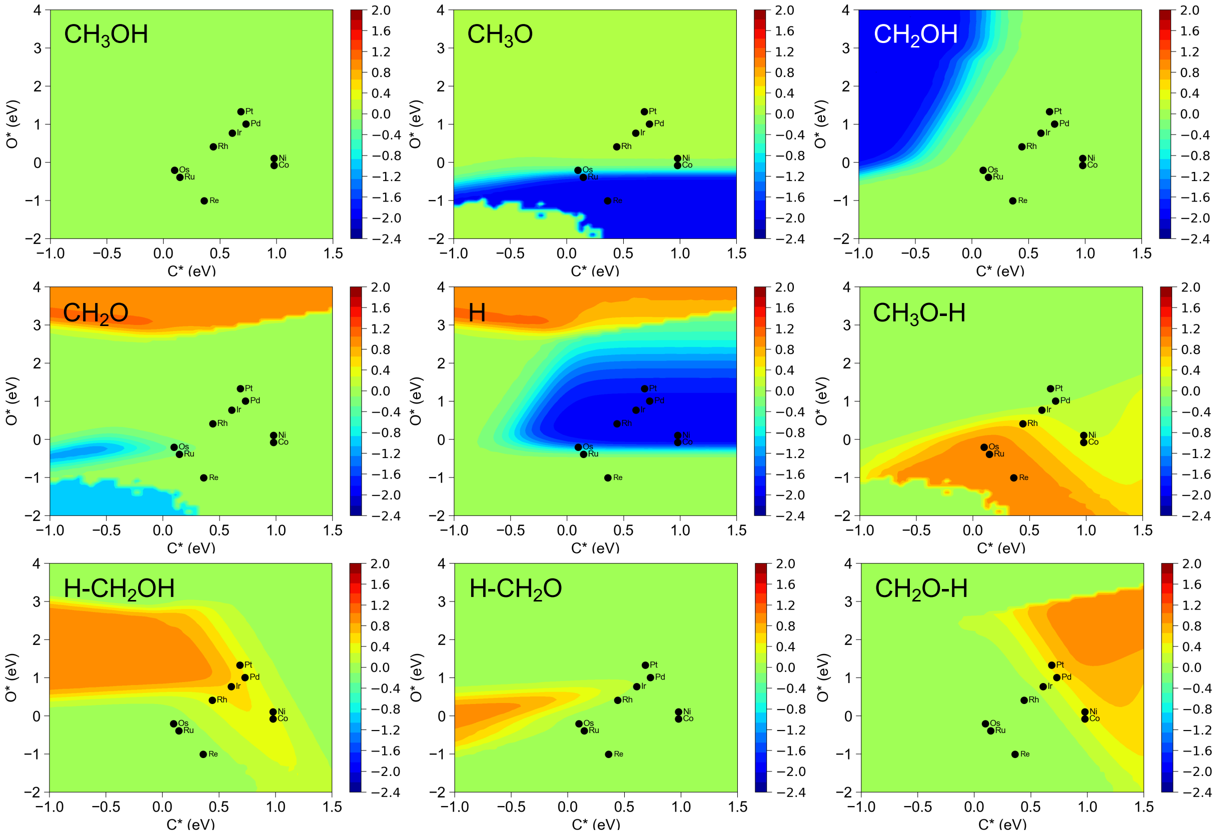

Supplementary Figure 12. Representative HR-TEM images and particle size distributions of Ni, Co, Pd, Pt and Ru catalysts supported over Al_2O_3. The term dp [3,2] refers to surface weighted mean particle size.

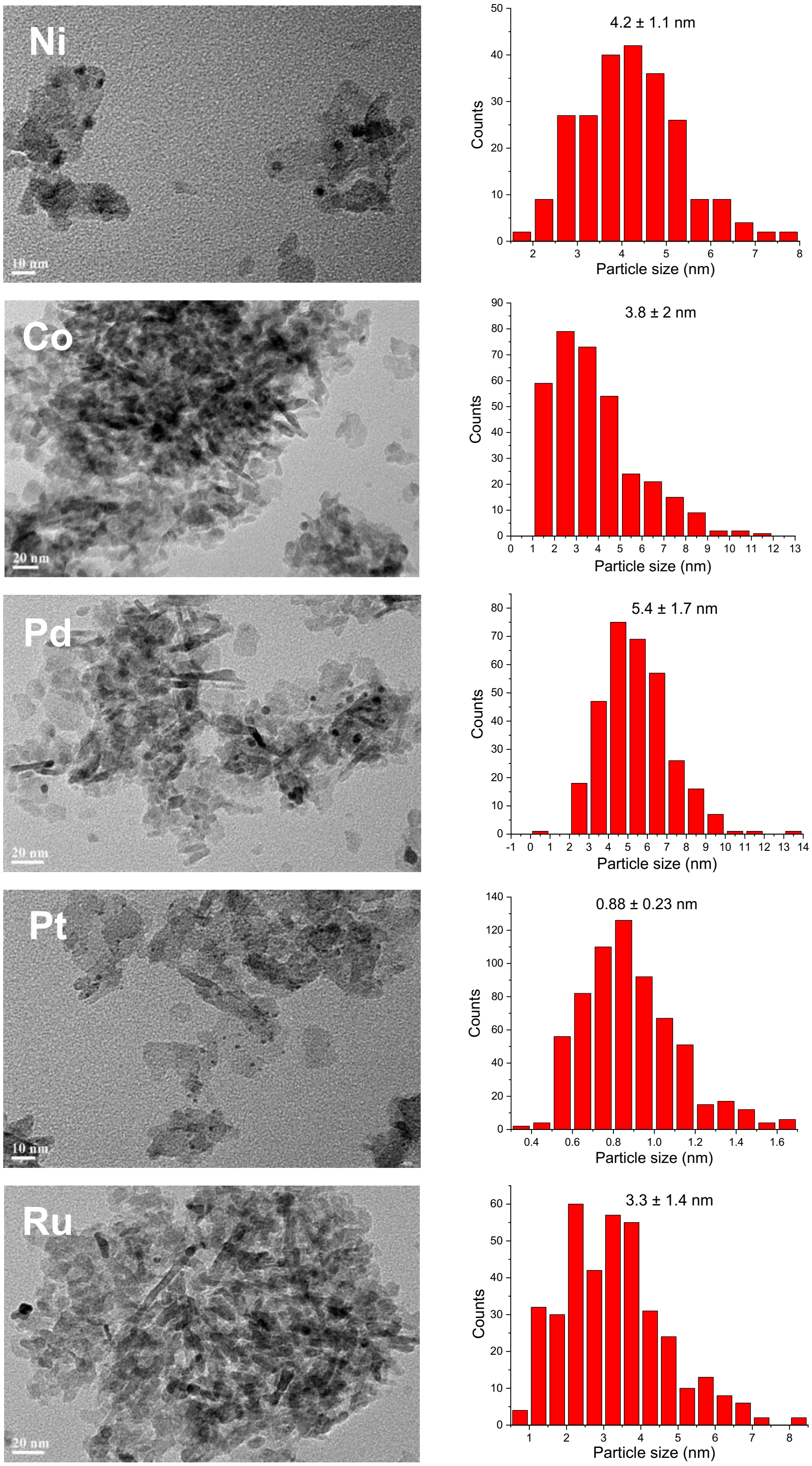

Supplementary Figure 13. Representative HR-TEM images and particle size distributions of Pd and Pt catalysts supported over SiO_2. The term dp [3,2] refers to surface weighted mean particle size.

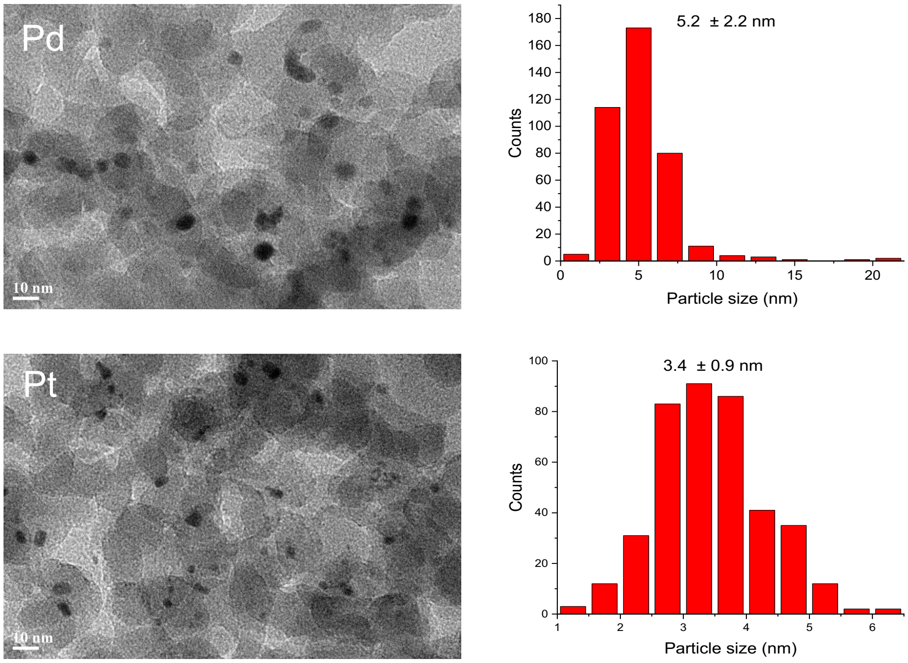

Supplementary Figure 14. Selectivity of 2-octanone and 1-octene over alumina-supported metal catalysts at comparable 2-octanol conversion (28-47%). Reaction conditions: T = 453 K, P = 101 kPa, p_{OL} = 14-20 kPa, p_{H2} = 44 kPa, $WHSV_{OL}$ = 3.3-32 h^{-1}. The catalysts were pre-reduced during 4 h at different temperatures according to the corresponding H_2-TPR profiles (**Supplementary Figure 16**): 453 K for Pd/Al_2O_3, 473 K for Pt/Al_2O_3 and Ru/Al_2O_3, 723 K for Co/Al_2O_3, 773 K for Ni/Al_2O_3.

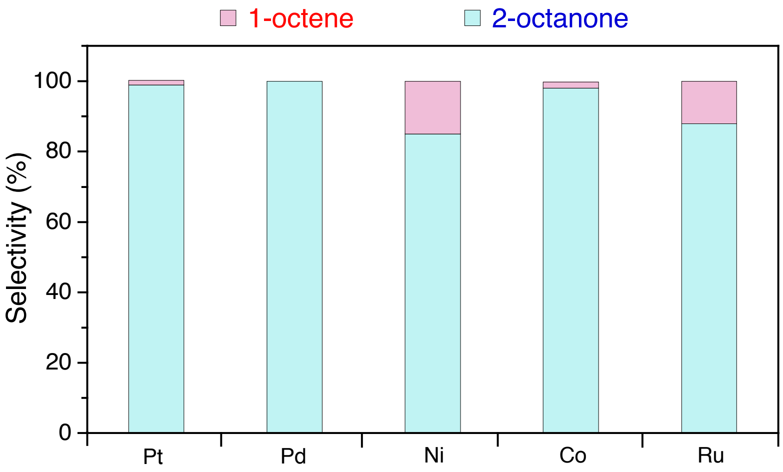

Supplementary Figure 15. TG profiles on the spent metal-supported catalysts.

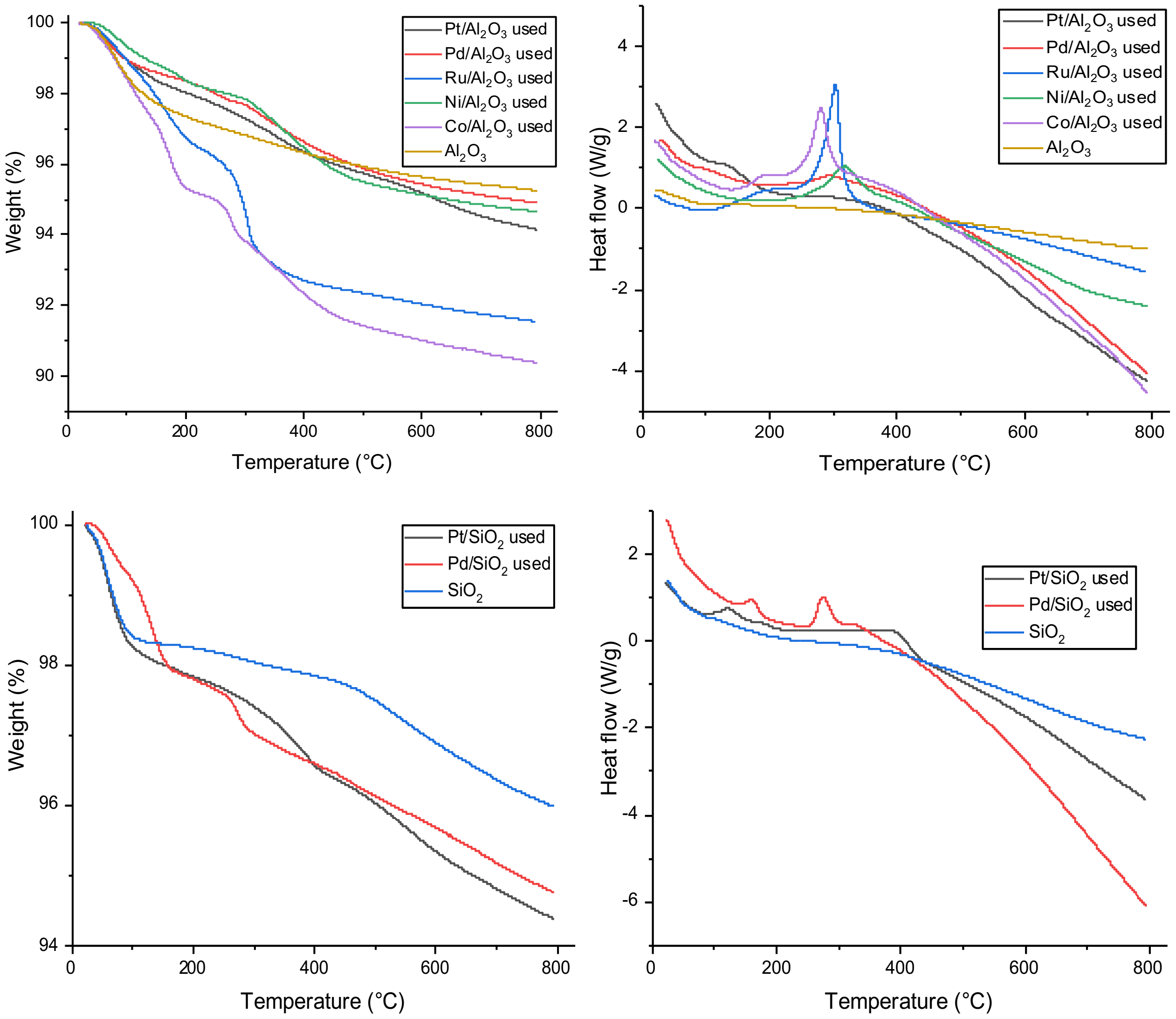

Supplementary Figure 16. H_2-TPR profiles of the different metal-supported catalysts.

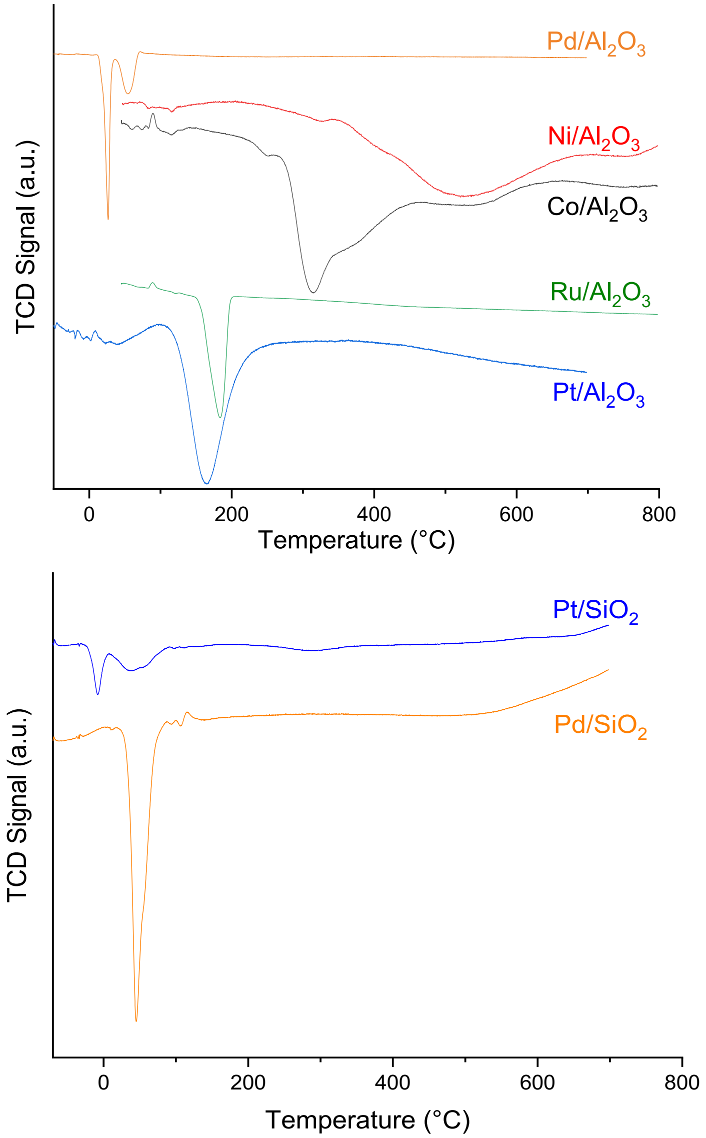

Supplementary Figure 17. Schematic surface structures of dilute alloys of one metal B atom in metal A (a), and two metal B atoms in A (b) on the close-packed surface.

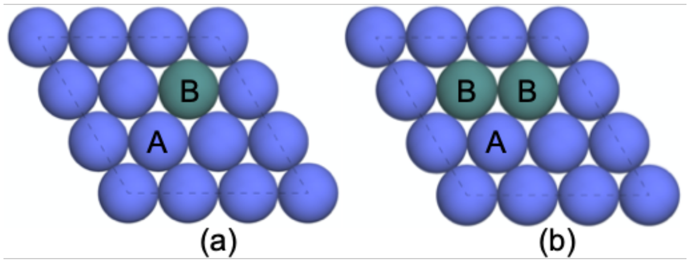

Supplementary Figure 18. The scaling relations between adsorption energies of C and CH_2OH as well as O and CH_3O on transition metals and Mo_2N surfaces.

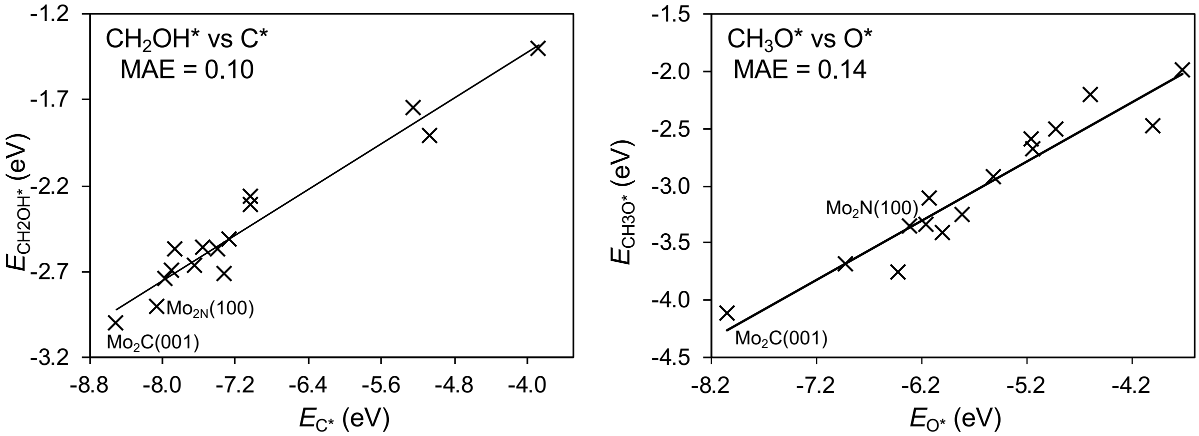

Supplementary Figure 19. XRD pattern of the as-prepared Mo_2N sample.

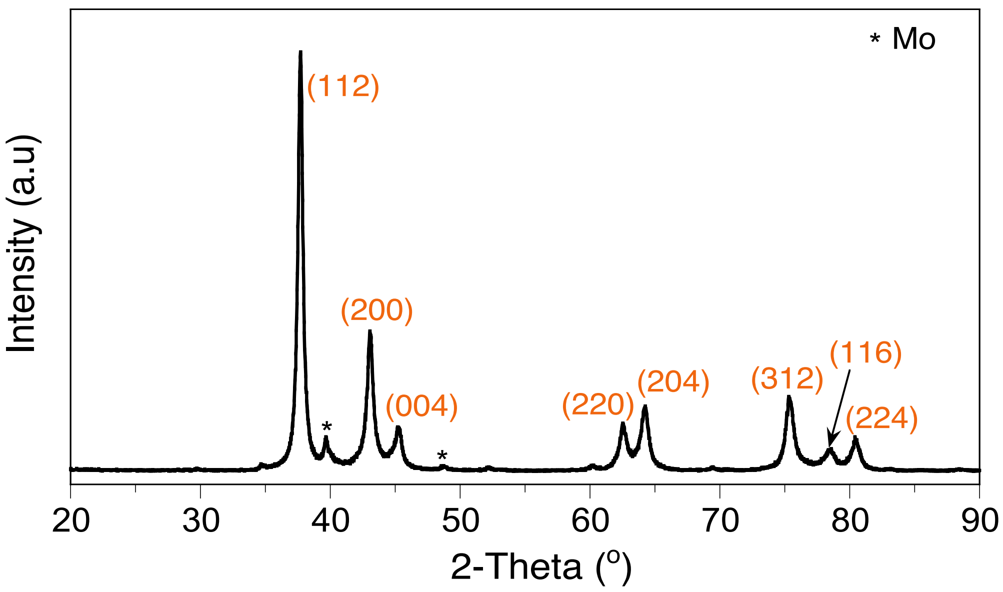

Supplementary Figure 20. HR-TEM micrographs of the as-prepared Mo_2N sample showing the location of (112), (200) and (004) planes, and particle size distribution

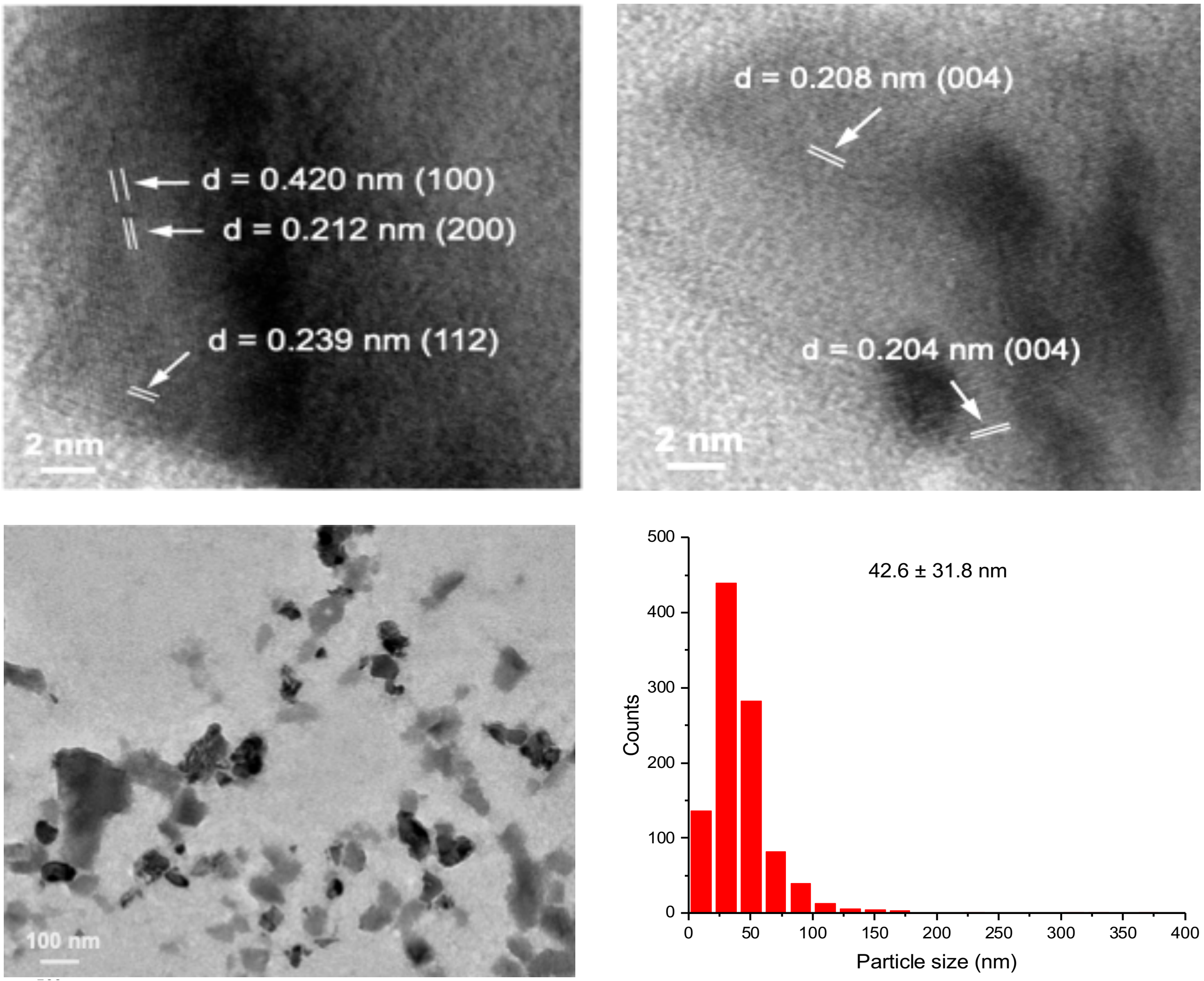

Supplementary References

1 Kresse, G.; Furthmüller, J. *Comput. Mater. Sci.* **1996**, *6*, 15-50.

2 Kresse, G.; Furthmüller, J. *Phys. Rev. B* **1996**, *54*, 11169-11186.

3 Blochl, P. E. *Phys. Rev. B* **1994**, *50*, 17953-17979.

4 Kresse, G. *Phys. Rev. B* **1999**, *59*, 1758-1775.

5 Perdew, J. P.; Burke, K.; Ernzerhof, M. *Phys. Rev. Lett.* **1996**, *77*, 3865-3868.

6 Methfessel, M.; Paxton, A. T. *Phys. Rev. B* **1989**, *40*, 3616-3621.

7 Steinmann, S. N.; Corminboeuf, C. *J. Chem. Theory Comput*. **2011**, *7*, 3567-3577.

8 Henkelman, G.; Jónsson, H. *J. Chem. Phys*. **2000**, *113*, 9978-9985.

9 Medford, A. J.; Shi, C.; Hoffmann, M. J.; Lausche, A. C.; Fitzgibbon, S. R.; Bligaard, T.; Nørskov, J. K. CatMAP: a Software Package for Descriptor-based Microkinetic Mapping of Catalytic Trends. Catal. Lett. 2015, 145, 794-807.

10 Campbell, C. T. *Top. Catal*. **1994**, 1, 353.

11 Campbell, C. T. *J. Catal*. **2001**, 204, 520.

12 Stegelmann, C.; Andreasen, A.; Campbell, C. T. *J. Am. Chem. Soc*. **2009**, *131*, 8077–8082.

13 Cárdenas-Lizana, F.; Gómez-Quero, S.; Perret, N.; Kiwi-Minsker, L.; Keane, M.A. *Catal. Sci. Technol.*, **2011**,*1*, 794-801.

14 Borodzinski, A.; Bonarowska, M. *Langmuir* **1997**, *13*, 5513-5620.

15 Shiraishi, Y.; Fujiwara, K.; Sugano, Y.; Ichikawa, S.; Hirai, T. *ACS Catal*. **2013**, *3*, 312-320.
