# Supplementary material for: Identification of active catalysts for the acceptorless dehydrogenation of alcohols to carbonyls
Source: Nat Commun. 2021 Aug 24;12:5100. doi: 10.1038/s41467-021-25214-1 (PMC8385104; doi:10.1038/s41467-021-25214-1)
Supplement: Supplementary file 2 — Description of Additional Supplementary Files [file 41467_2021_25214_MOESM2_ESM.pdf]

Description of Additional Supplementary Files

File Name: Supplementary Data 1
Description: Coordinates of all optimized structures generated in this study
